# Prognostic value and immunological role of CSNK1D in human cancers

**DOI:** 10.18632/aging.205009

**Published:** 2023-09-08

**Authors:** Jianguo Wang, Baohong Hu, Weixing Wang

**Affiliations:** 1 Department of Hepatobiliary Surgery, Renmin Hospital of Wuhan University, Wuhan, Hubei 430060, PR China

**Keywords:** CSNK1D, cancer, HCC, prognosis, immune infiltration

## Abstract

CSNK1D, also known as CK1δ, is a crucial gene involved in various biological processes such as cell cycle, transcriptional regulation, apoptosis, cell polarity, and cell motility. It is associated with different cancers and neurodegenerative diseases. This study aimed to investigate the role of CSNK1D in multiple human cancers, particularly hepatocellular carcinoma (HCC), through *in vitro* experiments. The research utilized various online resources and databases like UCSC, NCBI, GEPIA2, HPA, cBioPortal, SangerBox, UALCAN, and TCGA for analyzing CSNK1D expression, prognosis significance, immune features, and gene alterations in cancers. RT-PCR was employed to evaluate CSNK1D expression in normal liver and liver cancer cell lines. *In vitro* experiments, including CCK-8, Edu, wound healing, and Transwell assays, were conducted to assess CSNK1D’s biological function in HCC cells. Results demonstrated consistent upregulation of CSNK1D in various tumors. Heightened CSNK1D expression correlated with reduced overall survival and disease-free survival rates in different cancer patient cohorts. Significant associations were found between CSNK1D expression levels and immune cell infiltrations, immune checkpoint inhibitors, tumor mutation burden, and microsatellite instability across multiple malignancies. Notably, statistical analyses using TCGA and ICGC data identified CSNK1D as a robust and independent prognostic biomarker in HCC. Inhibiting CSNK1D expression effectively hindered cell proliferation, migration, and invasion in cellular experiments. In conclusion, this study suggests that CSNK1D may serve as a biomarker for tumor prognosis and immunotherapy. It influences the proliferation and metastasis of HCC cells.

## INTRODUCTION

Cancer is a global public health concern responsible for millions of deaths annually. Despite significant advancements in cancer research, our comprehension of the complete spectrum of genetic mutations that drive cancer initiation and progression remains limited. Cancer is widely recognized as a heterogeneous disease, and various subtypes exhibit distinctive genetic and molecular traits. Therefore, it is imperative to understand comprehensively the genetic alterations underlying cancer to develop effective therapeutic interventions [1, 2].

CSNK1D is a gene that encodes a protein and belongs to the CK1 family, also known as CK1δ. CSNK1D plays a crucial role in several biological processes, including cell cycle regulation, transcriptional regulation, apoptosis, cell polarity, and cell motility [3–6]. Furthermore, CSNK1D is closely associated with the development of multiple human diseases, such as cancer and neurodegenerative diseases [7, 8]. In terms of its role in cancer, researchers have made significant discoveries. Studies have shown that CSNK1D is essential in tumor cell proliferation, metastasis, angiogenesis, and drug resistance [9, 10]. Abnormal expression and functional defects of CSNK1D are closely associated with the development and progression of various cancers, including melanoma, breast, glioblastoma and colon cancers [11–14]. Additionally, some studies have revealed that CSNK1D plays a vital role in immunotherapy, and it could serve as a promising therapeutic target. CSNK1D can promote cancer development and progression through multiple mechanisms. For example, it can regulate various signaling pathways such as Wnt/β-catenin, PI3K/AKT/mTOR, and Hippo pathways to stimulate tumor cell proliferation and metastasis [5, 15, 16]. Furthermore, CSNK1D can also regulate tumor cell metabolism, including glucose and lipid metabolism, affecting tumor cell growth and survival [17, 18]. Therefore, CSNK1D is a potential valuable prognostic marker and therapeutic target for tumors.

Our objective for this study was to thoroughly scrutinize the variations in CSNK1D found in cancer, utilizing the Cancer Genome Atlas (TCGA) and other datasets accessible to the public. Our exploration encompassed analyzing the expression of CSNK1D mutations across various cancer types and their probable correlation with clinical results. Furthermore, we delved into the consequential meaning of CSNK1D mutations by examining a liver cancer cell line. To sum up, our research yields innovative revelations regarding the significance of CSNK1D in cancer and emphasizes its potential as a target for therapeutic interventions.

## MATERIALS AND METHODS

### Data collection

We retrieved transcriptome and clinical data for 33 tumors from the UCSC Xena website (https://xenabrowser.net/datapages/), in addition to single-nucleotide mutation data and calculated the tumor mutation burden. Furthermore, we obtained transcriptome data and clinical information for liver cancer from the ICGC database (https://dcc.icgc.org/).

### Differential and prognostic analysis

In order to investigate the differential expression of CSNK1D in normal and tumor tissues, we employed the “limma” R package and the Wilcoxon rank-sum test to analyze the transcriptome data of 33 tumors. Additionally, we evaluated the variance in CSNK1D expression between tumor tissues and adjacent normal tissues using the Xiantao tool (https://www.xiantao.love/) by integrating data from TCGA and GTEx. The median expression value of CSNK1D was used to generate Kaplan-Meier survival curves, utilizing the “SurvMiner” and “Survival” R packages. The relationship between CSNK1D expression and multiple human tumors survival rate was then examined using the “forestplot” R package [19]. To explore the correlation between CSNK1D expression and tumor stage, we employed UALCAN (https://ualcan.path.uab.edu/index.html).

### Correlation analysis

To examine the interplay between CSNK1D expression and the tumor microenvironment across various cancers, we leveraged the ESTIMATE function within the SangerBox tool (http://vip.sangerbox.com/home.html). This tool facilitated the assessment of stromal score, immune score, and ESTIMATE score. In order to delve deeper into the correlation between CSNK1D expression and immune cell infiltration, we employed seven computational tools, including EPIC, TIMER, IPS, QUANTIAEQ, xCell, MCPcounter, and CIBERSORT [20]. Furthermore, we investigated the relationship between CSNK1D expression and immune checkpoint, EMT-related, apoptosis-related, and autophagy-related molecules in 33 tumor types using the “limma,” “reshape2,” and “RColorBrewer” packages. We utilized TCGA data and the ‘fmsb’ R package to gain insights into the relationship between CSNK1D expression, tumor mutation burden (TMB), and microsatellite instability (MSI). Finally, we probed the mutation status of CSNK1D across multiple cancers by accessing the TIMER and cBioPortal databases.

### Protein network construction and gene enrichment analysis

To predict genes with similar functions to CSNK1D, we used the GeneMANIA database. We also performed gene ontology (GO) and Kyoto Encyclopedia of Genes and Genomes (KEGG) analysis on potential molecules with a role in CSNK1D using the “clusterProfiler” R package.

### Correlation analysis between CSNK1D expression and clinical characteristics

We examined the link between CSNK1D expression and clinical staging parameters, such as age, gender, pathological stage, tumor (TMN) stage, and histological grade in hepatocellular carcinoma. To assess the prognostic significance of CSNK1D in patients with liver cancer, we performed univariate and multivariate COX regression as well as receiver operating characteristic (ROC) analysis. Furthermore, we developed a nomogram that integrated the expression value of CSNK1D and key clinical characteristics, including age, gender, grade, and stage, to enable clinical prognostic evaluations of CSNK1D in hepatocellular carcinoma. Calibration curves were utilized to verify the accuracy of the nomogram’s prediction.

### Cell culture

The human liver cell line LO2 and HCC cell lines HCC-LM3, Hep-G2, MHCC-97H, and PLC/PRF/5 were obtained from the American Type Culture Collection (ATCC, Manassas, VA, USA) and maintained in a 37°C, 5% CO2 incubator. The human target gene CSNK1D short hairpin RNA (siRNA) was procured from Guangzhou RiboBio Co., Ltd. (https://www.ribobio.com/) with the following sequences:

genOFFTM st-h-CSNK1D_001: GTCGCATCGAATACATTCA
genOFFTM st-h-CSNK1D_002: GAGAGAGCGGAAAGTGAGT
genOFFTM st-h-CSNK1D_003: GGATTAGCGAGAAGAAAAT

For transfection, Hep-G2 and MHCC-97H cells were seeded in six-well plates and cultured in a CO2 incubator at 37°C until they adhered to the walls. Transfection was performed with lipofectamine 2000.

### Quantitative real-time PCR

Total RNA was extracted using TRIzol reagent (Thermo Fisher Scientific) and cDNA was generated using the Thermo Fisher Scientific kit. Quantitative real-time PCR was performed using the Quantagene q225 real-time PCR system with the following primers:

CSNK1D Forward Primer: CAGGAGAAGAGGTTGCCATCA
Reverse Primer: CAAGCAGCAGGACGGTTTTG
Beta action Forward Primer: TGATGGTGGGAATGGGTCAG
Reverse Primer: GGTGTGGTGCCAGATCTTCT

### CCK-8 assay

To assess cell viability, liver cancer cells (*N* = 3000) were transfected with either the silencing sequence of the target gene or a control sequence and cultured in 96-well plates. A 10% working solution of CCK8 (Abclonal, China) was added to each well, followed by incubation at 37°C for 2 hours. The relative cell viability was determined using spectrophotometry at a wavelength of 450 nm.

### EdU assay

When the cell density reached 30–50% in a six-well plate, cells were treated with Edu working solution for 2 h, fixed with 4% paraformaldehyde for 20 min, washed with PBS three times, and permeabilized with 0.3% Triton X-100 for 15 min. The BeyoClick™ EdU Cell Proliferation Kit (Alexa Fluor 488) (https://www.beyotime.com/index.htm) was used to complete the reaction, and images were captured using a microscope.

### Wound healing assay

To perform this experiment, cells were allowed to grow until they reached almost 100% confluence in a 6-well plate. Subsequently, a 200 μL pipette tip was used to create a scratch in the cell layer. The cells were then washed with low serum media to avoid interference with cell growth. Images were captured at 0 h, 24 h, and 48 h after scratching using a microscope, allowing for the observation of the healing process over time.

### Transwell migration and invasion assay

First, 5 × 10^4^ cells were seeded in 200 μL serum-free medium and added to the upper chamber of a Transwell, while 600 μL of complete medium was added to the lower chamber. The Transwell was then incubated at 37°C with 5% CO2 for 24 hours. After incubation, the cells were fixed with 4% paraformaldehyde for 20 minutes and stained with crystal violet for 30 minutes. Before imaging under a microscope, the upper chamber was gently wiped with sterile cotton and air-dried at room temperature. The only difference between the invasion and migration assays was the presence of a layer of 100 μL of extracellular matrix gel in the upper chamber, mixed with serum-free medium at a ratio of 1:8.

### Statistical analysis

We performed statistical analyses to compare gene expression differences using the Wilcoxon rank sum test and Kruskal-Wallis test. To evaluate the correlation between the two groups, we employed Spearman or Pearson correlation analysis. For assessing survival characteristics, we utilized the Kaplan-Meier method and Cox regression analysis. The chi-square test and Fisher’s exact test were applied to analyze clinical characteristics. All statistical analysis and visualization were conducted using R software and GraphPad Prism 9. Significance levels were represented as *P* < 0.05 (^*^), *P* < 0.01 (^**^), and *P* < 0.001 (^***^).

### Data availability

The original contributions presented in the study are included in the article/Supplementary Figure. Further inquiries can be directed to the corresponding author.

## RESULTS

### The CSNK1D expression in multiple human tumors

**Figure 1:**
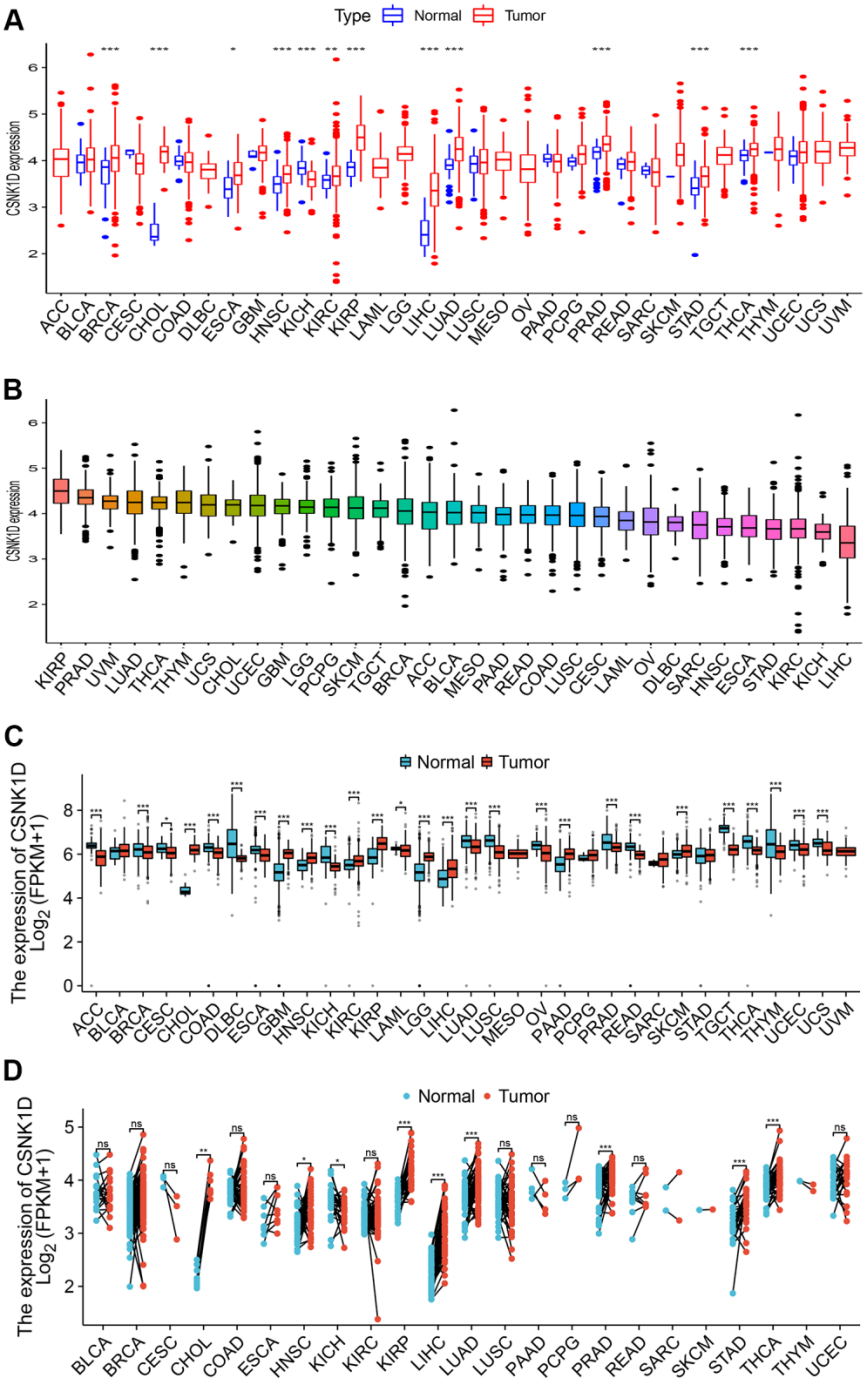
**The expression level of CSNK1D in multiple human cancers.** (**A**) CSNK1D expression levels in different cancers with TCGA data. (**B**) The expression levels of CSNK1D in cancers were sorted from highest to lowest. (**C**) CSNK1D expression levels in different cancers with TCGA combined with GTEx database. (**D**) The paired comparison analysis of expression differences of CSNK1D in tumor and corresponding adjacent tissues. ns, *p* ≥ 0.05. ^*^p < 0.05; ^**^p < 0.01; ^***^p < 0.001. ^****^p < 0.0001.

In this study, we evaluated the expression differences of CSNK1D across 33 types of tumors using RNA-seq data from TCGA alone, TCGA combined with GTEx, and paired data from 18 tumors. As shown in Figure 1A–1C, CSNK1D exhibited high expression levels in BRCA, CHOL, COAD, ESCA, HNSC, KICH, KIRC, KIRP, LIHC, LUAD, PRAD, STAD, and THYM. Furthermore, paired comparison analysis between tumor and corresponding normal tissues revealed that CSNK1D was significantly overexpressed in BRCA, CHOL, ESCA, HNSC, KICH, KIRC, KIRP, LIHC, LUAD, STAD, and THCA (Figure 1D).

### Prognostic evaluation of CSNK1D in multiple cancers

**Figure 2:**
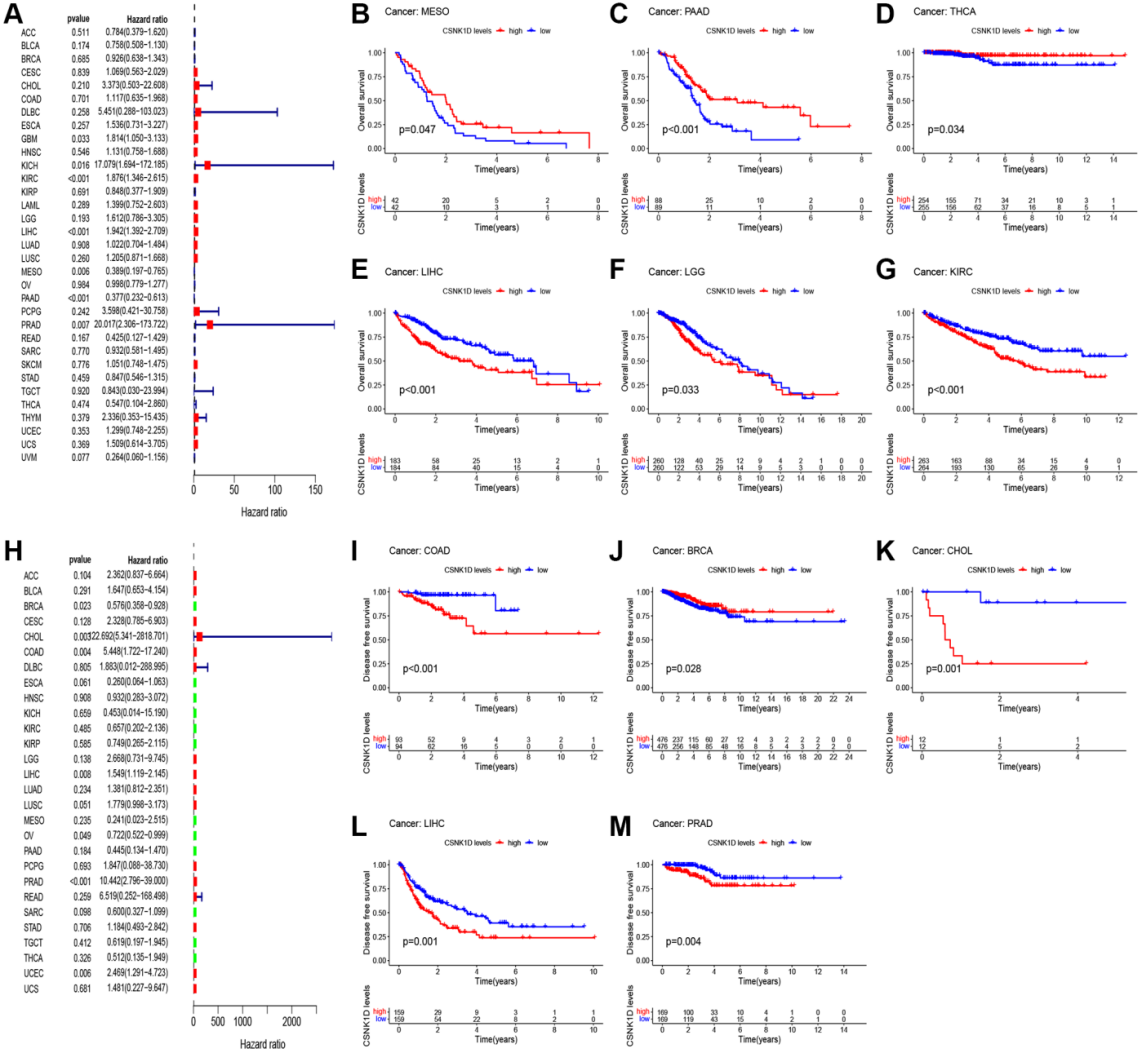
**Relationship between CSNK1D expression and patient prognosis.** (**A**) Correlation between the expression of CSNK1D and overall survival (OS) in multiple tumor types based on TCGA cohort. (**B**–**G**) Difference in the OS between the CSNK1D high and low expression groups. (**H**) Correlation between the expression of CSNK1D and disease-free survival (DFS) in multiple tumor types based on TCGA cohort. (**I**–**M**) Difference in the DFS between the CSNK1D high and low expression groups.

In this section, we aimed to investigate the impact of CSNK1D expression on the prognosis of cancer patients using TCGA RNA-seq and clinical data. We employed univariate Cox regression analysis to explore the association between CSNK1D expression and overall survival (OS) in 33 types of cancer. Our findings showed that CSNK1D was a poor prognostic factor in GBM, KICK, KIRC, LIHC, and PRAD, while it acted as a protective factor in MESO and PAAD (Figure 2A). Similarly, Kaplan-Meier survival analysis indicated that high CSNK1D expression was linked to poor OS in KIRC, LGG, and LIHC, and to favorable OS in MESO, PAAD, and THCA (Figure 2B–2G). Moreover, we performed univariate Cox regression analysis to investigate the correlation between CSNK1D expression and disease-free survival (DFS), disease-specific survival (DSS), and progression-free survival (PFS) in the 33 types of cancer (Figure 2H, Supplementary Figure 1A, 1J). Subsequently, we carried out corresponding Kaplan-Meier survival analysis to validate the results. Our analysis showed that high CSNK1D expression was associated with worse DFS in COAD, CHOL, LIHC, and PRAD, while it had a positive effect in BRCA (Figure 2I–2M). Furthermore, Kaplan-Meier survival analysis of disease-specific survival (DSS) demonstrated that high CSNK1D expression was associated with worse prognosis in LIHC, PRAD, LUSC, LGG, and KIRC, while it had a beneficial effect in MESO, PAAD, and THCA (Supplementary Figure 1B–1I). Finally, Kaplan-Meier survival analysis of progression-free survival (PFS) revealed that high CSNK1D expression was linked to worse prognosis in LIHC, COAD, PRAD, and STAD, while it had a positive effect in PAAD (Supplementary Figure 1K–1O). Overall, our results suggest that CSNK1D expression plays a critical role in cancer prognosis, and its effects may vary depending on the cancer type.

### Multiple tumors analysis of association between CSNK1D and clinicopathology

**Figure 3:**
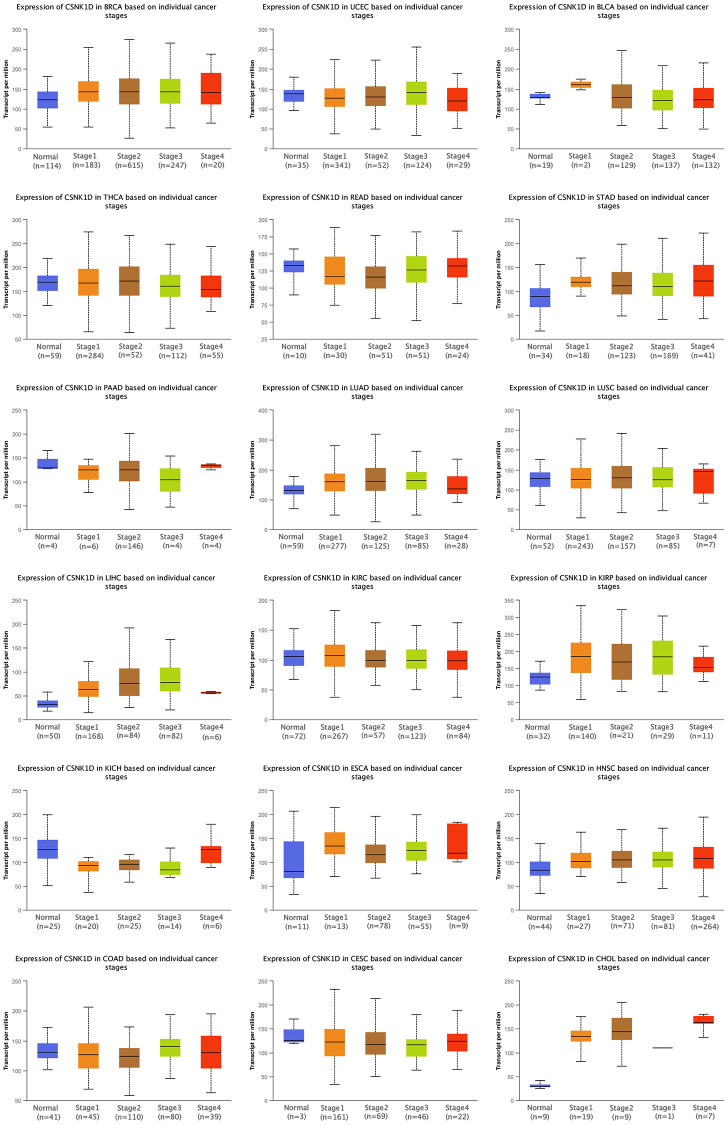
**Clinical correlation analysis of CSNK1D expression in pan-caner.** ns, *p* ≥ 0.05; ^*^p < 0.05; ^**^p < 0.01; ^***^p < 0.001. ^****^p < 0.0001.

We utilized the ULCAN online platform to investigate the correlation between CSNK1D expression and clinical characteristics of multiple cancer types. Our findings revealed a significant increase in CSNK1D expression with the advancement of pathological stage in BRCA, CHOL, HNSC, KIRP, LIHC, LUAD, READ, and STAD, while a decrease was observed in KICH (Figure 3). Furthermore, we evaluated the expression disparities of CSNK1D based on Age, Gender, and Grade, and our results are displayed in Supplementary Figure 2. Our study sheds light on the potential role of CSNK1D as a crucial biomarker in cancer diagnosis and progression monitoring.

### Immune characteristics of CSNK1D in the tumor microenvironment

**Figure 4:**
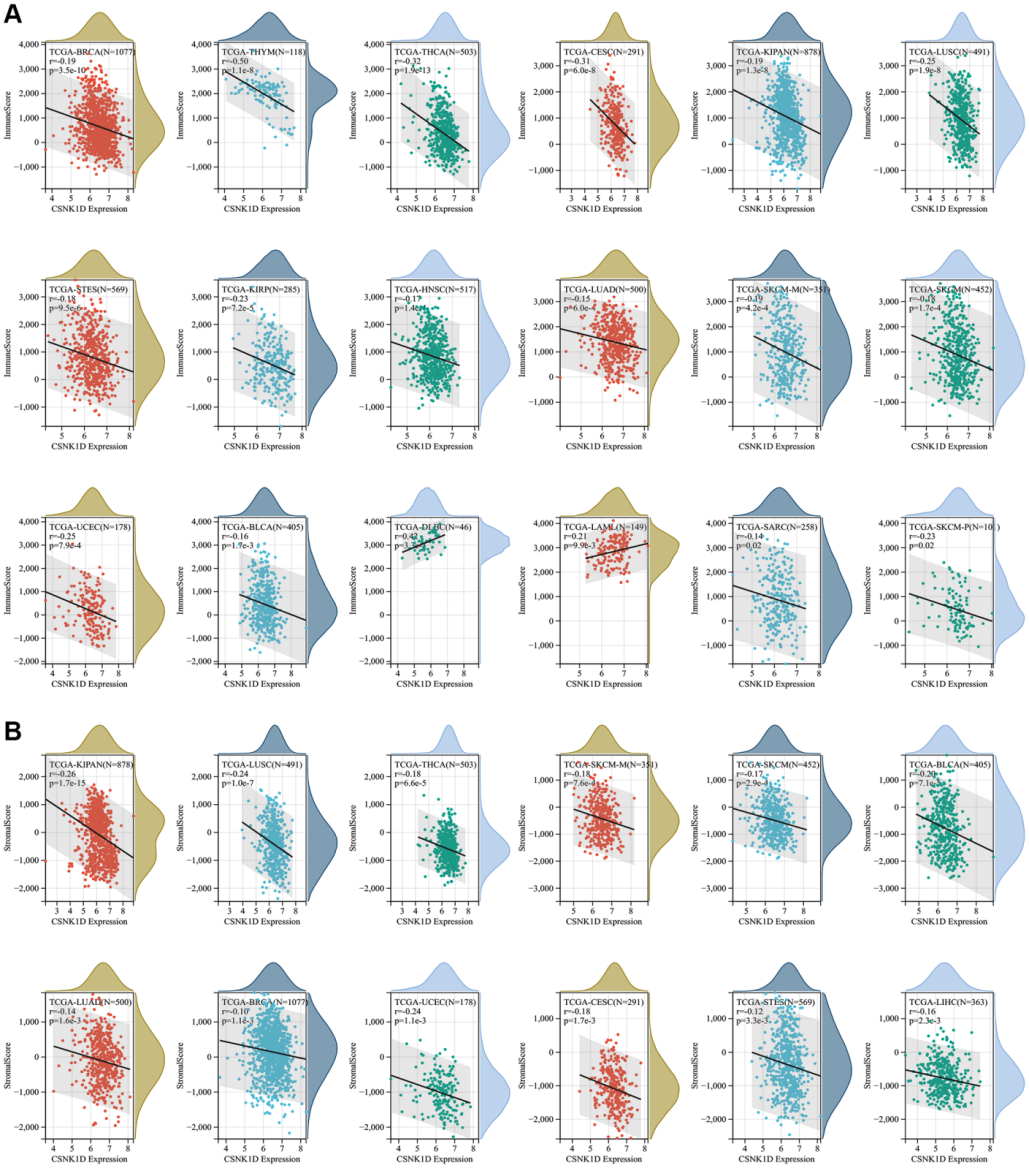
**Correlation of CSNK1D expression with immune score, and stromal score in various cancers.** (**A**) Correlation of CSNK1D expression with Immune Score. (**B**) Correlation of CSNK1D expression with Stromal Score.

The tumor microenvironment consists of various components, including immune cells, a mesenchymal environment, and a range of molecular factors both inside and outside the tumor. These factors play a crucial role in the initiation, progression, metastasis, and response to therapy of the tumor [21]. Firstly, our objective was to investigate the immune characteristics of CSNK1D in the tumor microenvironment (TME) across various cancers. To achieve this, we employed the ESTIMATE algorithm available on the SangerBox website. It enabled us to evaluate the correlation between CSNK1D expression and three scores (immune, stromal, and estimate) across 33 cancer types. Our findings revealed a negative correlation between CSNK1D expression and immune scores in several cancers, including BRCA, THYM, THCA, CESC, KIPAN, LUSC, STES, KIRP, HNSC, LUAD, SKCM-M, SKCM-P, SKCM, UCEC, BLCA, and SARC, whereas a positive correlation was observed in DLBC and LAML (Figure 4A; *P* < 0.05). Additionally, the stromal scores were negatively correlated with CSNK1D expression in all cancers (Figure 4B; *P* < 0.05). Furthermore, our analysis of the estimate score demonstrated a negative correlation with CSNK1D expression in nearly all cancer types, except for DLBC and LAML (Supplementary Figure 3; *P* < 0.05). These results highlight the potential importance of CSNK1D in shaping the immune characteristics of the TME in various cancers.

**Figure 5:**
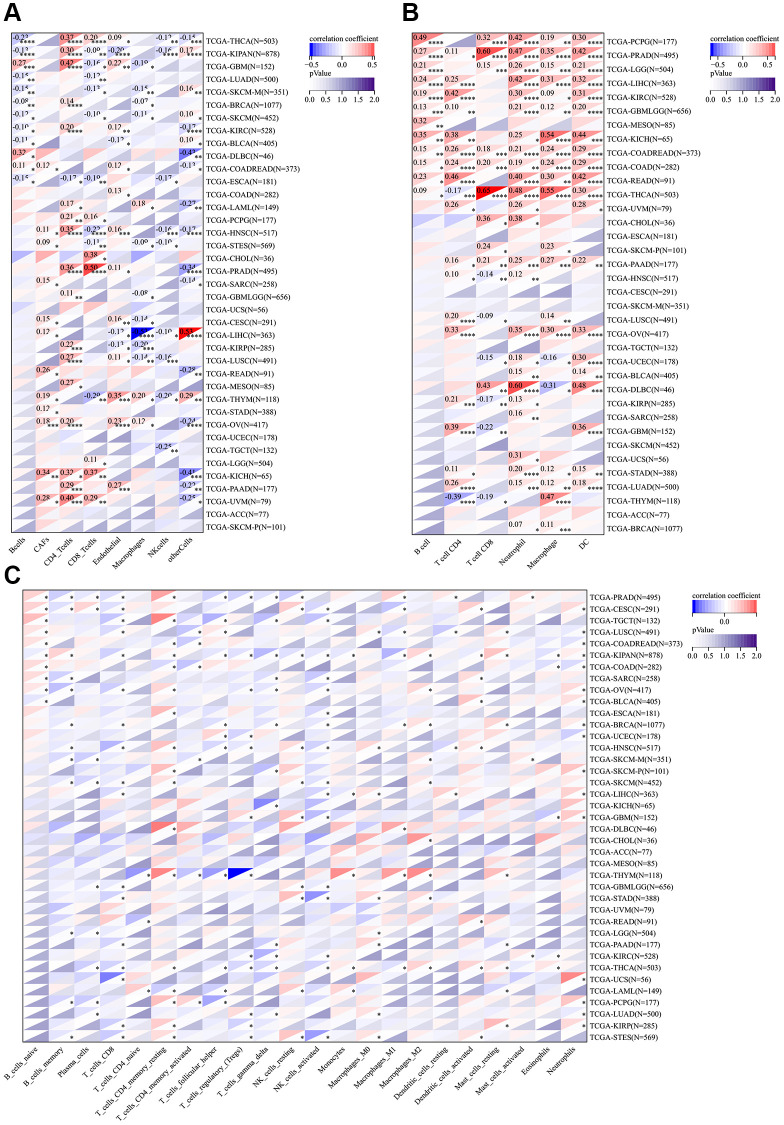
**Correlation analysis between the expression of CSNK1D and immune cell infiltration.** (**A**) The relationship between CSNK1D expression and immune cell infiltration by EPIC algorithm. (**B**) The relationship between CSNK1D expression and immune cell infiltration by TIMER algorithm. (**C**) The relationship between CDNK1D expression and immune cell infiltration by CIBERSORT algorithm. ^*^p < 0.05; ^**^p < 0.01; ^***^p < 0.001. ^****^p < 0.0001.

To investigate the relationship between CSNK1D and immune cells in various cancers, we utilized seven immune infiltration algorithms, namely EPIC, TIMER, IPS, QUANTIAEQ, xCell, MCPcounter, and CIBERSORT. Our results revealed a strong correlation between CSNK1D and stromal cells in the tumor microenvironment of these cancers. Notably, high CSNK1D levels were positively associated with CD4_Tcells, Neutrophils, and NK_cells in almost all cancer types. Moreover, we found that CSNK1D exhibited a significant correlation with diverse immune cells in LIHC, including Monocytic_lineage, Macrophages_M1, Macrophages_M2, and DC cells, indicating a strong positive correlation (Figure 5A–5C; Supplementary Figure 4).

**Figure 6:**
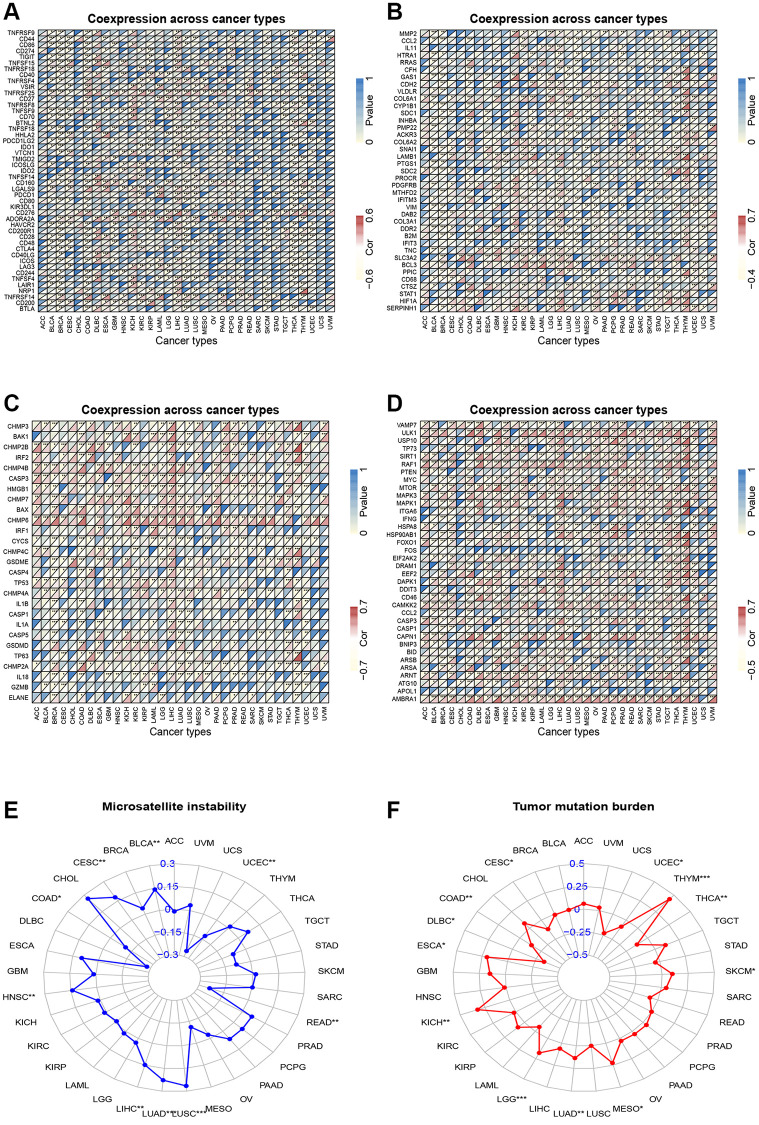
**Correlation analysis.** (**A**) Correlation analysis of CSNK1D expression and immune checkpoint inhibitors. (**B**) Correlation analysis between CSNK1D expression and EMT-related molecules. (**C**) Correlation analysis between CSNK1D expression and Pyroptosis-related molecules. (**D**) Correlation analysis between CSNK1D expression and Autophagy-related molecules. (**E**) Association between CSNK1D expression and MSI. (**F**) Association between CSNK1D expression and TMB. ^*^p < 0.05; ^**^p < 0.01; ^***^p < 0.001. ^****^p < 0.0001.

We also investigated the association between CSNK1D expression and immune checkpoint inhibitors in various cancers. Our results indicated a positive correlation between CSNK1D and most immune checkpoint inhibitors, with a particular emphasis on LIHC, DLBC, KICH, and ESCA. However, we found a significant negative correlation between CSNK1D and some immune checkpoints in THCA and BRCA. Additionally, CSNK1D showed a positive correlation with TNFRSF25 and CD276 in most cancers (Figure 6A). EMT is known to play a significant role in tumorigenesis, invasion, metastasis, and treatment resistance of tumors [22]. Therefore, we examined the correlation between CSNK1D expression and EMT-related molecules. Our findings revealed a positive correlation between CSNK1D expression and EMT molecules such as BCL3, SLC3A2, and LAMB1 in THYM, KICH, LIHC, GMB, and THCA. Interestingly, we observed a negative correlation between CSNK1D and HTRA12 in almost all types of cancers (Figure 6B). Pyroptosis is a recently identified inflammatory-related and programmed cell death pathway that is crucial for cancer progression and immune regulation [23]. Our analysis showed a positive correlation between CSNK1D expression and pyroptosis-related molecules, including CHMP4B, CHMP6, CHMP7, and CASP3, in most tumors (Figure 6C). Autophagy is a critical degradation and recycling system that promotes tumor growth and invasiveness [24]. Therefore, we investigated the relationship between CSNK1D and autophagy-related molecules. Our findings indicated a positive correlation between CSNK1D and almost all autophagy molecules in LIHC and YHYM (Figure 6D). Microsatellite instability (MSI) and tumor mutation burden (TMB) are emerging as biomarkers for immune therapy [25, 26]. Therefore, we examined the relationship between MSI/TMB and CSNK1D expression in multiple cancers. Our results showed a positive correlation between CSNK1D and MSI in most cancers, while it was negatively correlated in READ, UCEC, and COAD. Moreover, we observed a positive correlation between CSNK1D and TMB in YHYM, ESCA, KICH, LGG, LUAD, MESO, and SKCM, while it was negatively correlated with DLBC (Figure 6E, 6F).

### Genetic alteration of CSNK1D in multiple human cancers

**Figure 7:**
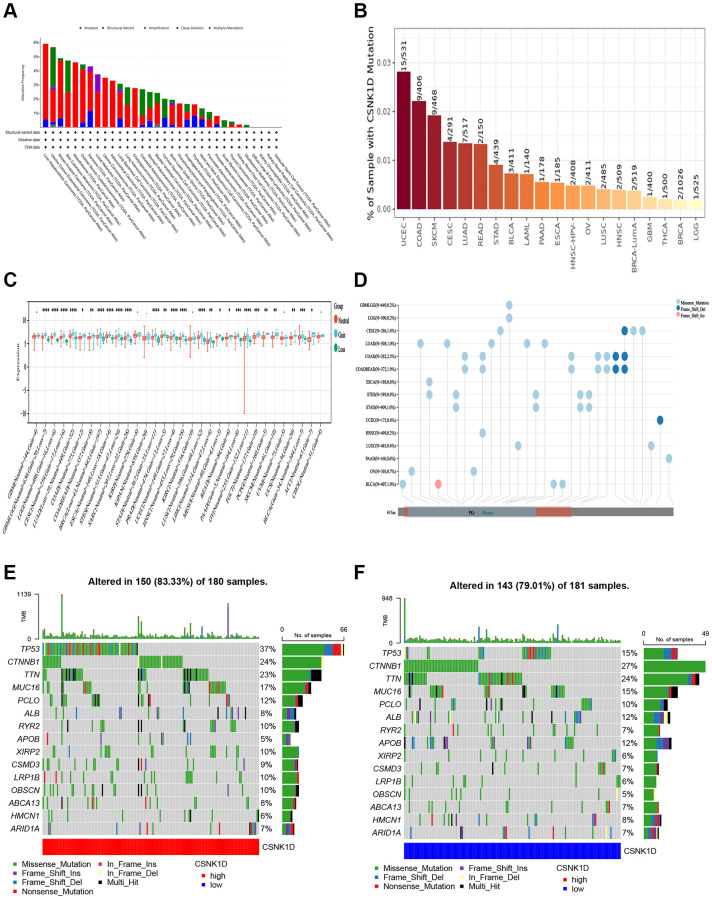
**Distinct genetic alteration profiles of CSNK1D in pan-cancer based on the TCGA database.** (**A**) Genetic alteration landscape (Mutation, Structural Variant, Amplification, and Deep Deletion) of CSNK1D in pan-cancer based on the TCGA database through the cBioPortal tool. (**B**) Mutation rate of CSNK1D among tumors by the TIMER tool. (**C**) CNVs of CSNK1D in various tumors by SangerBox portal. (**D**) SNVs of CSNK1D among different types of the tumor via SangerBox tool. (**E**) Tumor somatic mutation waterfall graph in CSNK1D high group based on TCGA-LIHC database. (**F**) Tumor somatic mutation waterfall graph in CSNK1D low group based on TCGA-LIHC database.

Genetic alterations, which can include mutations, deletions, or amplifications of oncogenes or tumor suppressor genes, have the potential to cause phenotypic changes that may trigger tumorigenesis and promote cancer progression. In this study, we analyzed the genetic changes in the CSNK1D gene using the TCGA cancer dataset through the cBioPortal tool, which included mutations, structural variations, amplifications, and deep deletions. Our findings revealed that liver hepatocellular carcinoma and uterine corpus endometrial carcinoma had the highest mutation rates of CSNK1D (>5%) (Figure 7A). We further examined the mutation rate of CSNK1D in cancer using the TIMER database, which showed that the mutation rate of CSNK1D was highest in UCSC (15/531) and COAD (9/406) (Figure 7B). To further explore the expression relationship between CSNK1D and copy number variations (CNVs) in different types of tumors, we utilized the SangerBox tool based on the TCGA database. We observed significant differences in most cancers, such as GBMLGG, LGG, CESC, LUAD, BRCA, STES, SARC, HNSC, LUSC, LIHC, OV, ACC, etc. (Figure 7C). Single nucleotide variations (SNVs) of the CSNK1D gene among tumors were also analyzed and presented in Figure 7D. Additionally, we analyzed the correlation between CSNK1D expression and specific genomic features in the TCGA-LIHC dataset. Our results indicated that the CSNK1D high group (*n* = 150) had higher somatic mutation frequencies of TP53 (37%), CTNNB1 (24%), and TTN (23%) genes, while the CSNK1D low group (*n* = 142) had higher somatic mutation frequencies of TP53 (15%), CTNNB1 (27%), and TTN (24%) genes (Figure 7E, 7F).

### Clinical correlation analysis of CSNK1D in hepatocellular carcinoma

**Figure 8:**
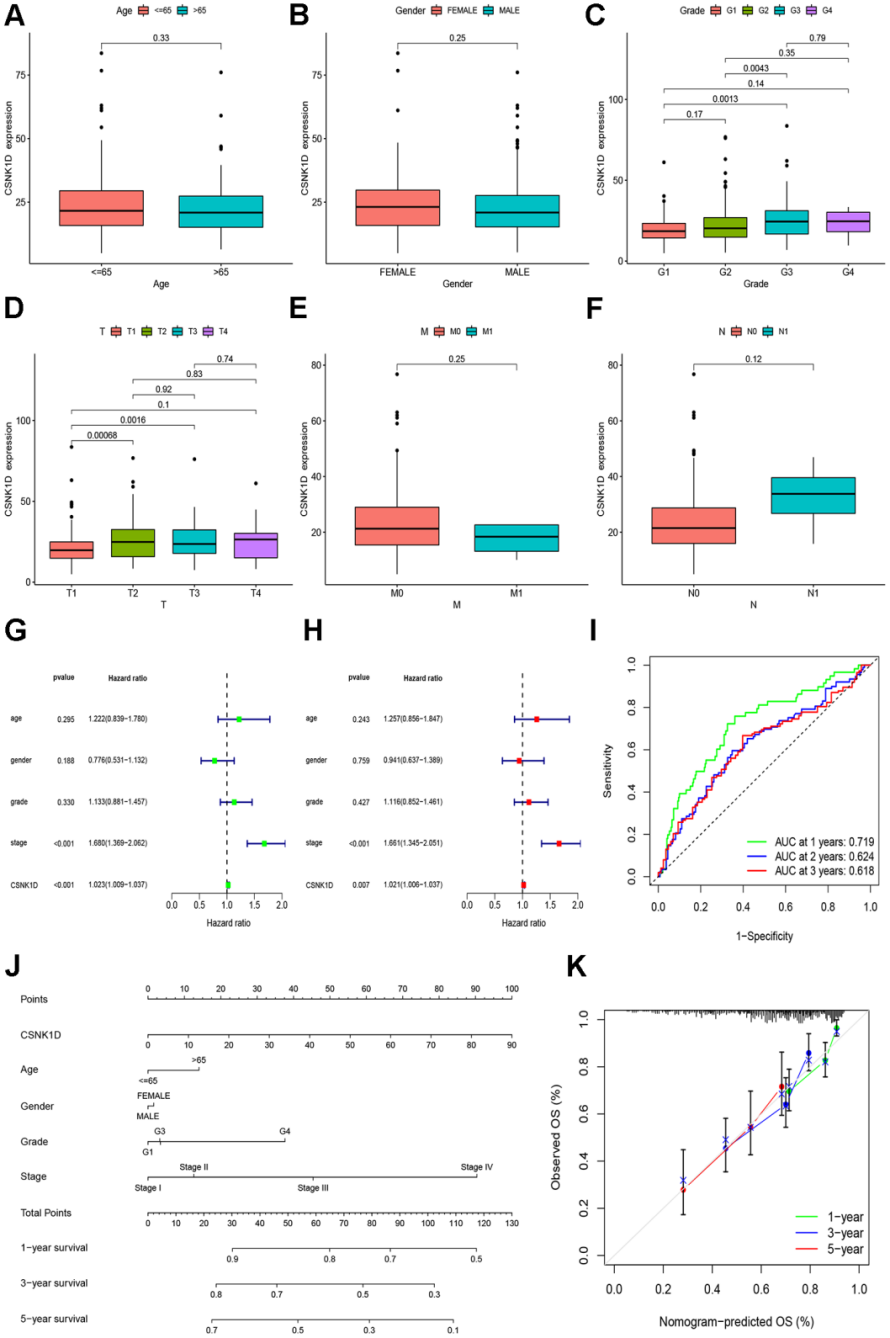
**Clinical correlation analysis of CSNK1D in hepatocellular carcinoma based on the TCGA-LIHC cohort.** Variation analysis of CSNK1D expression in different Ages (**A**), Gender (**B**), Grade (**C**), T stage (**D**), M stage (**E**), and *N* stage (**F**). (**G**, **H**) The prognostic significance of CSNK1D was analyzed by univariate and multivariate COX analysis. (**I**) Prognostic evaluation efficacy of CSNK1D by ROC. (**J**) A nomogram based on CSNK1D expression and stage, Gender, Age, and grade. (**K**) The calibration curves for 1, 3, and 5-year OS.

The previous research findings have suggested that CSNK1D may have a significant role in the development of hepatocellular carcinoma. To further understand this relationship, we examined the expression of CSNK1D in hepatocellular carcinoma patients at different clinical stages. Firstly, we obtained the expression data from TCGA-LIHC database, which consisted of 50 normal samples and 374 tumor samples. Specific clinical information is provided in Supplementary Table 1. Our results indicate that CSNK1D expression levels were higher in patients with advanced T staging and histological grading (Figure 8C, 8D), whereas no significant differences were observed with respect to age, gender, N, and M staging (Figure 8A, 8B, 8E, 8F). These findings suggest that CSNK1D expression may be associated with the malignant progression of hepatocellular carcinoma. To evaluate the prognostic effect of CSNK1D, we conducted univariate and multivariate COX regression analyses, which revealed that CSNK1D is an independent prognostic factor for hepatocellular carcinoma (Figure 8G, 8H). Furthermore, the prognostic evaluation effect of CSNK1D was assessed using the ROC liver cancer overall survival rate. Our results demonstrate that CSNK1D has a good prognostic evaluation effect in hepatocellular carcinoma, with an AUC of 0.719, 0.624, and 0.618 for 1-year, 3-year, and 5-year survival rates, respectively (Figure 8I). To evaluate the clinical application of CSNK1D, we constructed a nomogram based on the expression of CSNK1D and pathological staging (Figure 8J). The accuracy of the prognostic evaluation model was assessed using calibration curves, and the results indicated that the nomogram was very accurate, almost as good as the ideal model (Figure 8K).

**Figure 9:**
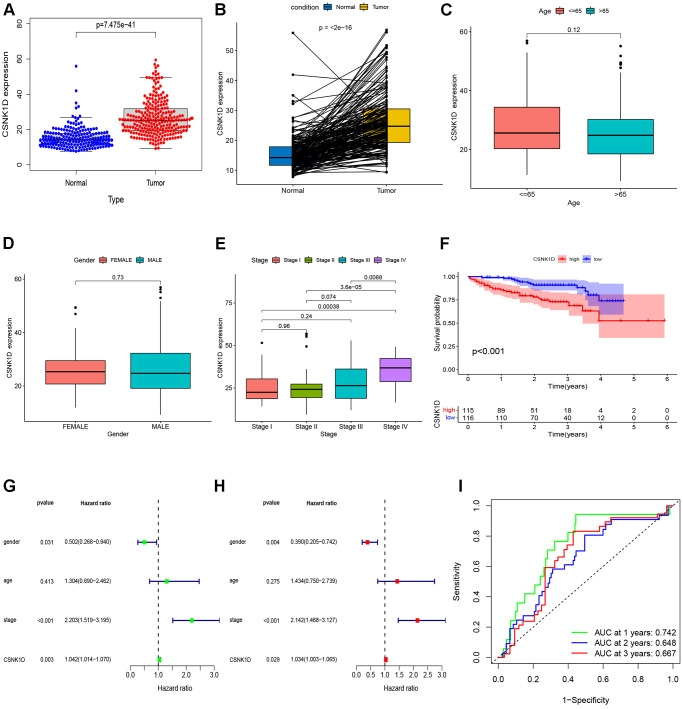
**Validation of CSNK1D expression in hepatocellular carcinoma based on the ICGC-LIRI-JP cohort.** (**A**) The mRNA expression of CSNK1D in normal and tumor. (**B**) Comparing the CSNK1D expression in paired normal and tumor. Variation analysis of CSNK1D expression in different Ages (**C**), Gender (**D**), stage (**E**). (**F**) K-M survival curves of patients with OS. (**G**, **H**) The prognostic significance of CSNK1D was analyzed by univariate and multivariate COX analysis. (**I**) Prognostic evaluation efficacy of CSNK1D by ROC.

To provide further confirm the prognostic value of CSNK1D in hepatocellular carcinoma (HCC), we analyzed the expression of CSNK1D in both cancerous and adjacent tissues from the ICGC-LIRI-JP cohort. To begin, we collected the expression data and clinical information for ICGC-LIRI-JP from the ICGC database. The dataset included 202 normal samples and 240 tumor samples, ensuring a robust sample size for analysis. Detailed clinical information can be found in Supplementary Table 2. Our analysis, including non-paired (Figure 9A) and paired (Figure 9B) comparisons, indicated higher expression of CSNK1D in liver cancer tumor tissues. We further evaluated the relationship between CSNK1D expression in HCC and clinical staging, and found that the expression of CSNK1D increased with increasing stage, but was not related to age and gender (Figure 9C–9E). Additionally, Kaplan-Meier curve analysis showed that patients with high CSNK1D expression had poorer survival outcomes (Figure 9F). Furthermore, both univariate and multivariate COX analysis indicated that CSNK1D was an independent prognostic risk factor for HCC (Figure 9G, 9H). To assess the prognostic effect of CSNK1D, we performed ROC survival rate analysis for HCC and found that CSNK1D exhibited a good prognostic evaluation effect, with AUCs of 0.742, 0.648, and 0.667 for 1 year, 3 years, and 5 years, respectively (Figure 9I).

Furthermore, to strengthen the evidence regarding the prognostic implications of CSNK1D expression in liver cancer, we conducted an analysis using the liver cancer dataset GSE14520 obtained from the GEO database. This dataset consisted of 241 normal samples and 247 samples from individuals diagnosed with cancer. Supplementary Table 3 contains additional specific clinical information. Our initial findings revealed that CSNK1D exhibited elevated expression levels in cancer patients (Supplementary Figure 5A). Moreover, we performed Kaplan-Meier survival analysis to assess the overall survival (OS) and relapse-free survival (RFS). The results clearly indicated that high expression of CSNK1D was associated with a poorer prognosis (Supplementary Figure 5B, 5C). Additionally, we explored the relationship between CSNK1D expression and clinical staging in hepatocellular carcinoma (HCC). Interestingly, we observed significant differences in CSNK1D expression levels in relation to alpha-fetoprotein (AFP) levels. However, we did not find any correlation between CSNK1D expression and factors such as age, gender, ALT, or Main Tumor Size (Supplementary Figure 5D–5I). We also validated the expression of CSNK1D in HCC using the HPA database and found that it was significantly upregulated in HCC compared to normal liver tissues (Supplementary Figure 6A, 6B). To further confirm this, we performed RT-PCR to detect the mRNA expression level of CSNK1D in normal liver cell line LO2 and four HCC cell lines (HCC-LM3, Hep-G2, MHCC-97H, and PLC/PRF/5), and found that the mRNA expression level of CSNK1D was higher in HCC cells (Supplementary Figure 6C). Taken together, our findings suggest that CSNK1D expression levels may serve as a valuable prognostic biomarker for hepatocellular carcinoma patients.

### Molecular interaction network and enrichment analysis

**Figure 10:**
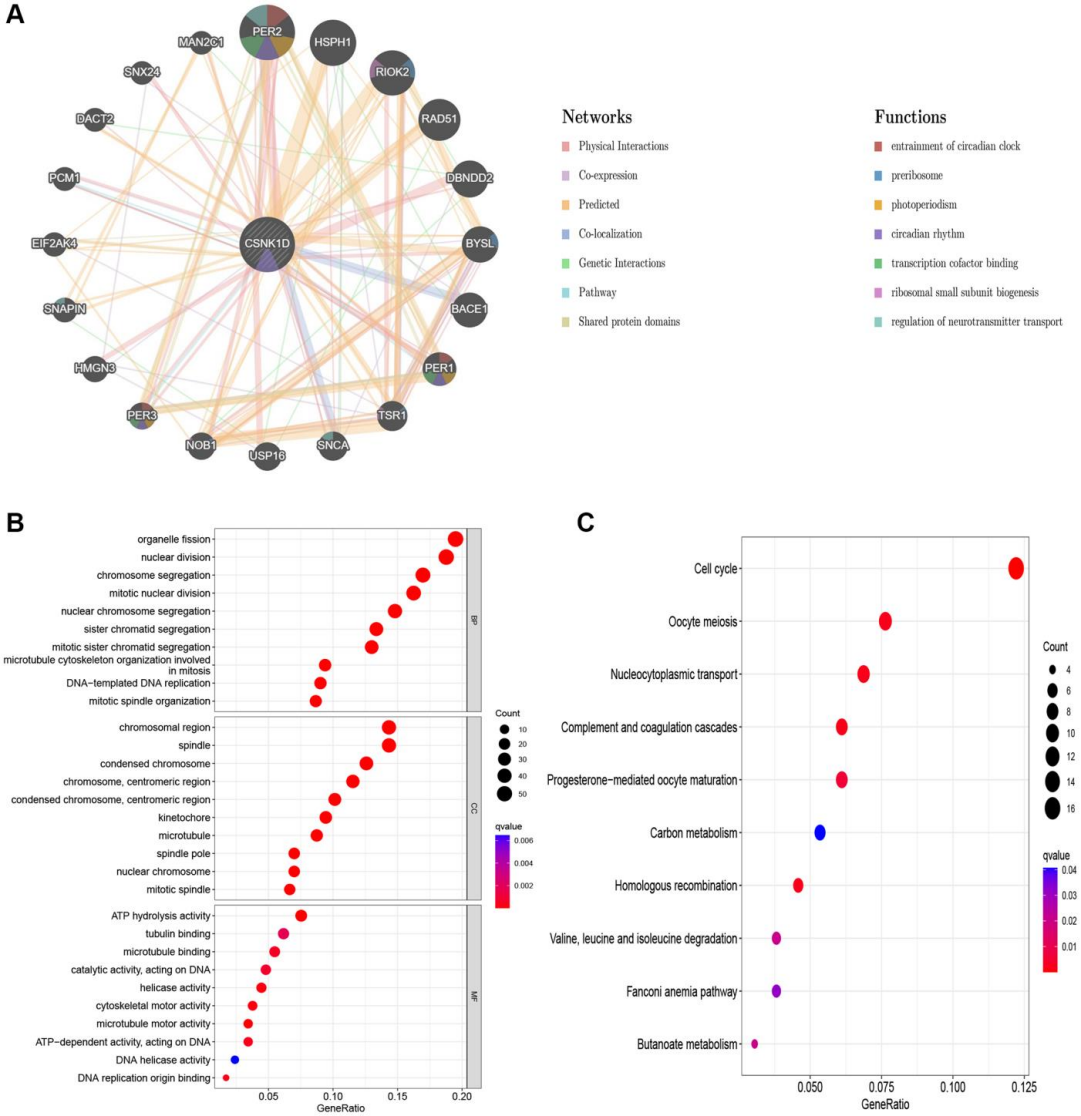
**Predicted function of CSNK1D.** (**A**) The potential interaction molecular network of CSNK1D by the GeneMANIA tool. (**B**, **C**) GO and KEGG functional enrichment analysis of CSNK1D.

The molecular interactions of CSNK1D and its potential roles were studied using GeneMANIA. Figure 10A shows the GeneMANIA network graph, which reveals that CSNK1D is mainly involved in several functions, including entrainment of the circadian clock, photoperiodism, preribosome, circadian rhythm, and transcription cofactor binding. CSNK1D interacts with various proteins such as PER2, PER3, PER1, RIOK2, TSR1, SNCA, USP16, NOB1, and others. To further explore the potential role of CSNK1D, KEGG and GO enrichment analysis were conducted. The biological process enrichment analysis revealed that these molecules are mainly involved in organelle fission, nuclear division, chromosome segregation, and nuclear chromosome segregation. The cellular component enrichment analysis revealed that these molecules are mainly associated with chromosomal regions, condensed chromosomes, and kinetochores. Furthermore, molecular function enrichment analysis showed that these molecules primarily participate in microtubule binding, tubulin binding, and ATP hydrolysis activity (Figure 10B). The KEGG analysis indicated that these molecules are mainly involved in the cell cycle, nucleocytoplasmic transport, and other pathways (Figure 10C).

### The effects of CSNK1D on proliferation, migration, and invasion of hepatocellular carcinoma cells

**Figure 11:**
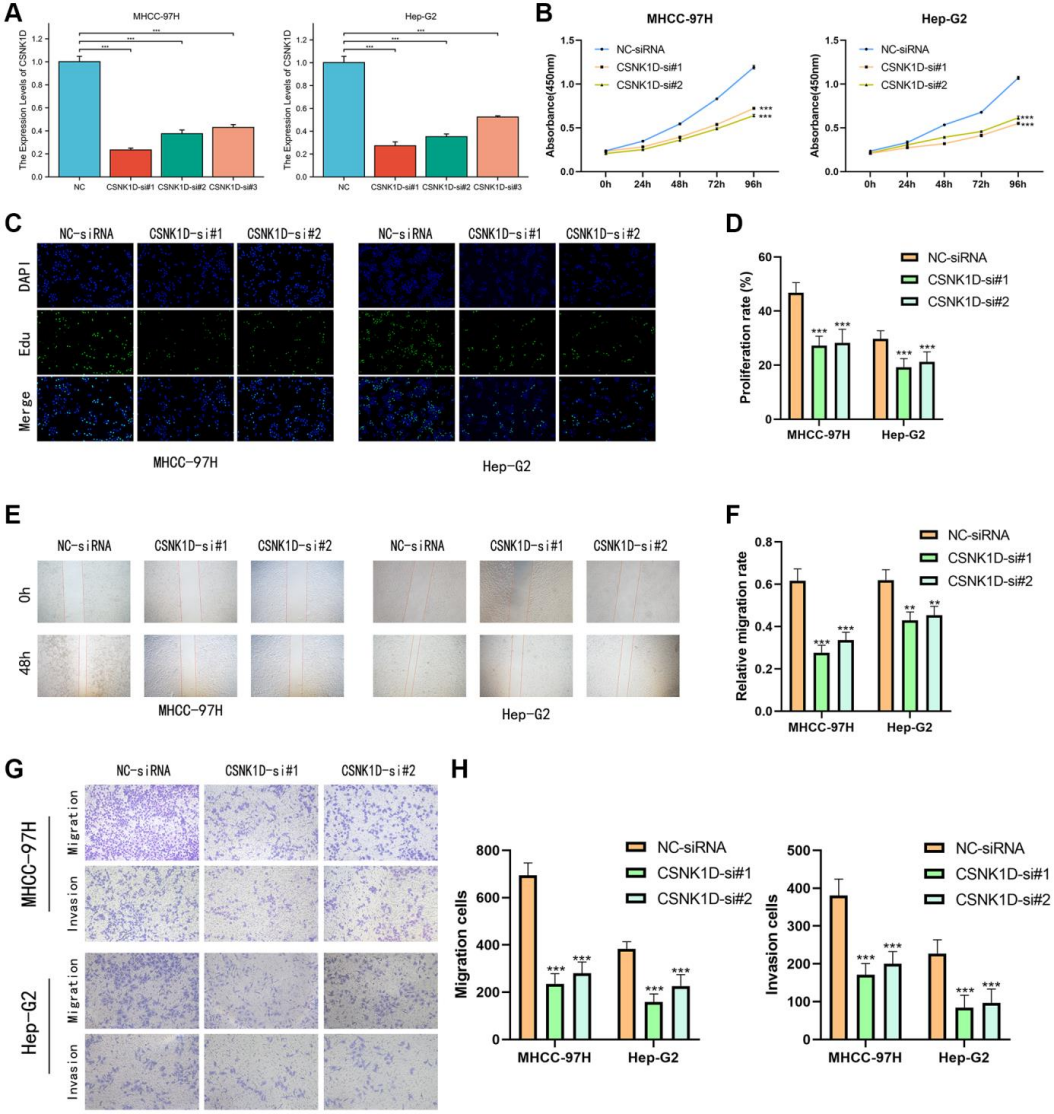
**CSNK1D downregulation inhibits hepatocellular carcinoma proliferation and invasion *in vitro*.** (**A**) The effects of transfection of Si-RNA transduction in HCC cell lines were detected by PCR analysis. (**B**) CCK-8 assay was performed to test the cell viability and proliferation. (**C**, **D**) EDU assays were performed to measure the proliferation ability of MHCC-97H and Hep-G2. (**E**, **F**) The migrated ability of MHCC-97H and Hep-G2 was measured by wound healing assay. (**G**, **H**) Transwell assay was performed the migrated and invaded abilities in MHCC-97H and Hep-G2 cell lines. ^*^p < 0.05, ^**^p < 0.01, ^***^p < 0.001.

Based on previous research, it was discovered that overexpression of CSNK1D is linked to poor survival in hepatocellular carcinoma (HCC) patients. To delve deeper into the role of CSNK1D in the development of HCC, three siRNA sequences were utilized to knockdown CSNK1D in MHCC-97H and Hep-G2 cell lines. The transfection efficiency was confirmed by qRT-PCR analysis, which demonstrated successful downregulation of CSNK1D expression by all three siRNA sequences (Figure 11A). The control group was named Si-RNA, while the experimental groups were named CSNK1D-Si#1 and CSNK1D-Si#2. Cell viability was measured using a CCK-8 assay, revealing a significant reduction in cell viability in both cell lines upon knockdown of CSNK1D (Figure 11B). These results were validated through an EDU experiment, which demonstrated that downregulation of CSNK1D expression significantly inhibited the proliferation of MHCC-97H and Hep-G2 cells (Figure 11C, 11D). To evaluate the migration ability of Si-RNA and CSNK1D-Si#1 and CSNK1D-Si#2 groups in HCC cell lines, a wound healing assay was performed, which demonstrated that downregulation of CSNK1D significantly inhibited cell migration (Figure 11E, 11F). Transwell assay further confirmed these results, indicating that downregulation of CSNK1D significantly impaired the migration and invasion of MHCC-97H and Hep-G2 cells (Figure 11G, 11H). These findings suggest that CSNK1D positively regulates the proliferation and invasion of HCC cells.

## DISCUSSION

CSNK1D, a member of the CK1 (formerly known as casein kinase 1) family, was first isolated by Graves and colleagues in the early 1990s. The human gene that encodes CSNK1D is located on the long arm of chromosome 17 (17q25.3) [27, 28]. Over the past few decades, the role of CSNK1D in physiological and pathological conditions has been extensively investigated [29]. Previous research has demonstrated that CSNK1D is involved in multiple biological processes, including the cell cycle, gene transcription, and cell differentiation [30–32]. Furthermore, abnormal expression of the CSNK1D gene has been reported in various cancers, and it is considered an important cancer-related gene that participates in biological processes such as tumor cell proliferation, migration, and invasion [33, 34]. Additionally, the CSNK1D gene is implicated in the formation of resistance of tumor cells to anticancer drugs [35]. Thus, the CSNK1D gene has become a key focus of cancer research and provides a significant foundation for the development of novel cancer treatment strategies.

According to the findings of this study, CSNK1D is significantly overexpressed in most tumors when comparing the TCGA and GTEx cohorts, both in paired and unpaired analyses. However, the expression of CSNK1D in KICH tumor tissues is notably reduced. Additionally, the K-M survival analysis method was used to investigate the relationship between CSNK1D expression and overall survival (OS) and disease-specific survival (DSS). The results indicate that abnormally high expression of CSNK1D in LIHC, LUAD, ESCA, and KIRP tumors is associated with poor OS and DSS. It is worth noting that the tumor immune microenvironment plays a crucial role in tumor cell clearance and immune evasion, and the infiltration of immune cells in the tumor immune microenvironment is linked to tumor development and prognosis [36, 37]. The study has uncovered a significant correlation between the expression of CSNK1D and the infiltration of CD4_Tcells and NK_cells in various types of tumors. Remarkably, a strong positive association was discovered between CSNK1D and several immune cells, including Monocytic_lineage, macrophages_m1, macrophages_m2, and DC cells, in the context of LIHC. It is worth noting that immune checkpoints are vital immune regulators that play a critical role in maintaining immune homeostasis and preventing autoimmunity [38, 39]. It is intriguing to note that the study findings demonstrate a positive correlation between CSNK1D and the majority of immune checkpoint inhibitors, particularly in LIHC, DLBC, KICH, and ESCA. CSNK1D may be involved in modulating the tumor microenvironment, which is known to be critical in tumor onset, advancement, and metastasis. Specifically, the processes of epithelial mesenchymal cell transformation, necrosis, and autophagy are all connected to these phenomena [40–42]. It is worth noting that our research has revealed a noteworthy positive correlation between the expression of CSNK1D and molecules involved in various cancer-related pathways across multiple cancer types. CSNK1D expression has also been found to be closely linked with TMB and MSI in several types of cancer, indicating its potential as a diagnostic and prognostic biomarker for cancer. Furthermore, we conducted a thorough investigation into the molecules affected by CSNK1D, and developed a molecular interaction network. Notably, we observed strong interactions between CSNK1D and PER2 and HSPH1. Enrichment analysis revealed that pathways related to organelle fission, condensed chromosomes, ATP hydrolysis activity, and cell cycle were potentially influenced by CSNK1D, all of which play critical roles in tumor development, highlighting the significant involvement of CSNK1D in tumor progression. Our findings suggest that abnormal expression of CSNK1D is closely linked to the prognosis of liver cancer patients. Therefore, we further analyzed the relationship between CSNK1D expression and clinical pathological staging, and developed a nomogram to facilitate the use of CSNK1D in evaluating liver cancer prognosis. Finally, our *in vitro* experiments have demonstrated that knocking down CSNK1D significantly reduces the proliferation, migration, and invasion abilities of MHCC-97H and Hep-G2 cells.

Our research has revealed that CSNK1D displays abnormal expression patterns in various types of cancer and holds great significance in cancer prognosis. Furthermore, we have uncovered a correlation between CSNK1D expression and immune cell infiltration in the tumor microenvironment, suggesting its potential role in regulating tumor immunity. *In vitro* studies have revealed that CSNK1D serves as an oncogenic factor in hepatocellular carcinoma, highlighting its promising candidacy as a therapeutic target in the realm of cancer treatment. Overall, this study sheds light on the pivotal role of CSNK1D in cancer development and progression and provides novel insights into its potential use as both a diagnostic and therapeutic target for diverse forms of cancer.

## CONCLUSION

In conclusion, this study reveals the aberrant expression patterns of CSNK1D in various types of cancer and highlights its significant relevance to cancer prognosis. Furthermore, a correlation between CSNK1D expression and immune cell infiltration in the tumor microenvironment has been uncovered, suggesting its potential role in regulating tumor immunity. *In vitro* experiments have demonstrated the oncogenic role of CSNK1D in hepatocellular carcinoma, indicating its potential as a therapeutic target in cancer treatment. Overall, this research sheds light on the pivotal involvement of CSNK1D in cancer development and progression and provides novel insights into its potential as a diagnostic and therapeutic target for diverse forms of cancer.

## Supplementary Materials

Supplementary Figures

Supplementary Table 1

Supplementary Table 2

Supplementary Table 3
