# Supplementary material for: Prognostic value and immunological role of CSNK1D in human cancers
Source: Aging (Albany NY). 2023 Sep 8;15(17):8948–75. doi: 10.18632/aging.205009 (PMC10522368; doi:10.18632/aging.205009)
Supplement: Supplementary Figures [file aging-15-205009-s001.pdf]

SUPPLEMENTARY FIGURES

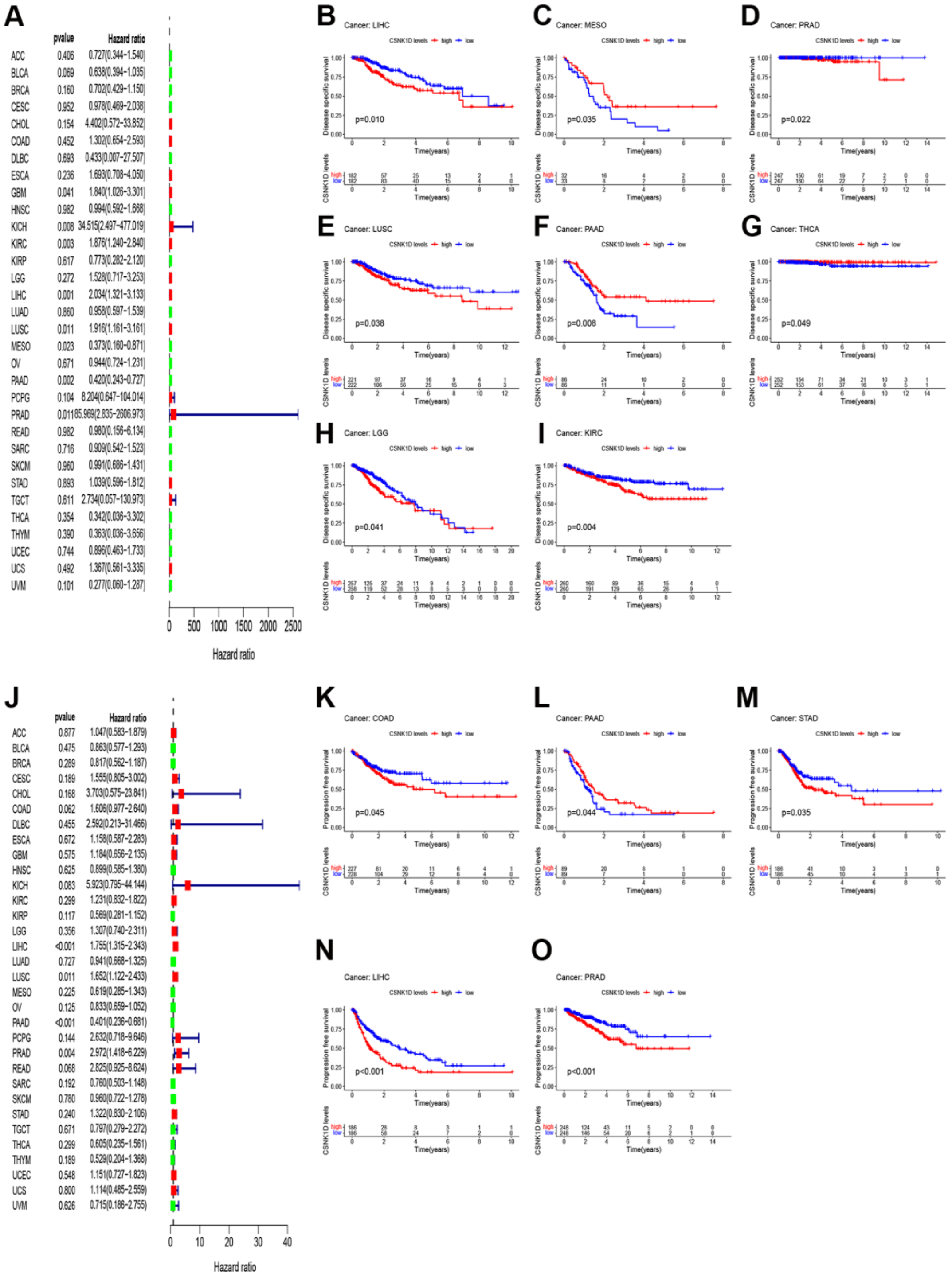

Supplementary Figure 1. Relationship between CSNK1D expression and patient prognosis. (**A**) Correlation between the expression of CSNK1D and disease-specific survival (DSS) in multiple tumor types based on TCGA cohort. (**B**–**I**) Difference in the DSS between the CSNK1D high and low expression groups. (**J**) Correlation between the expression of CSNK1D and progression-free survival (PFS) in multiple tumor types based on TCGA cohort. (**K**–**O**) Difference in the PFS between the CSNK1D high and low expression groups.

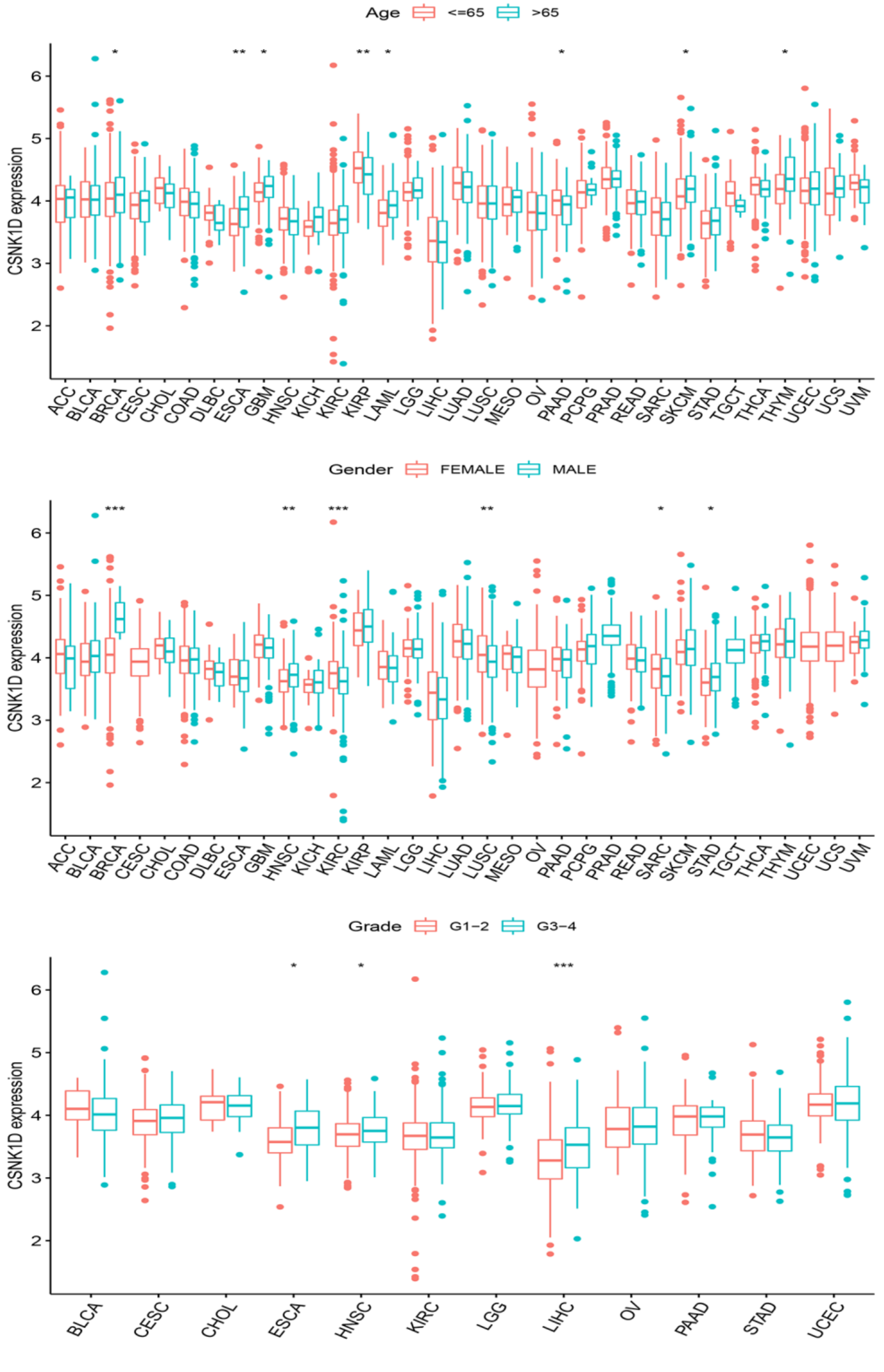

Supplementary Figure 2. Expression differences of CSNK1D in age, gender and grade based on TCGA cohort.

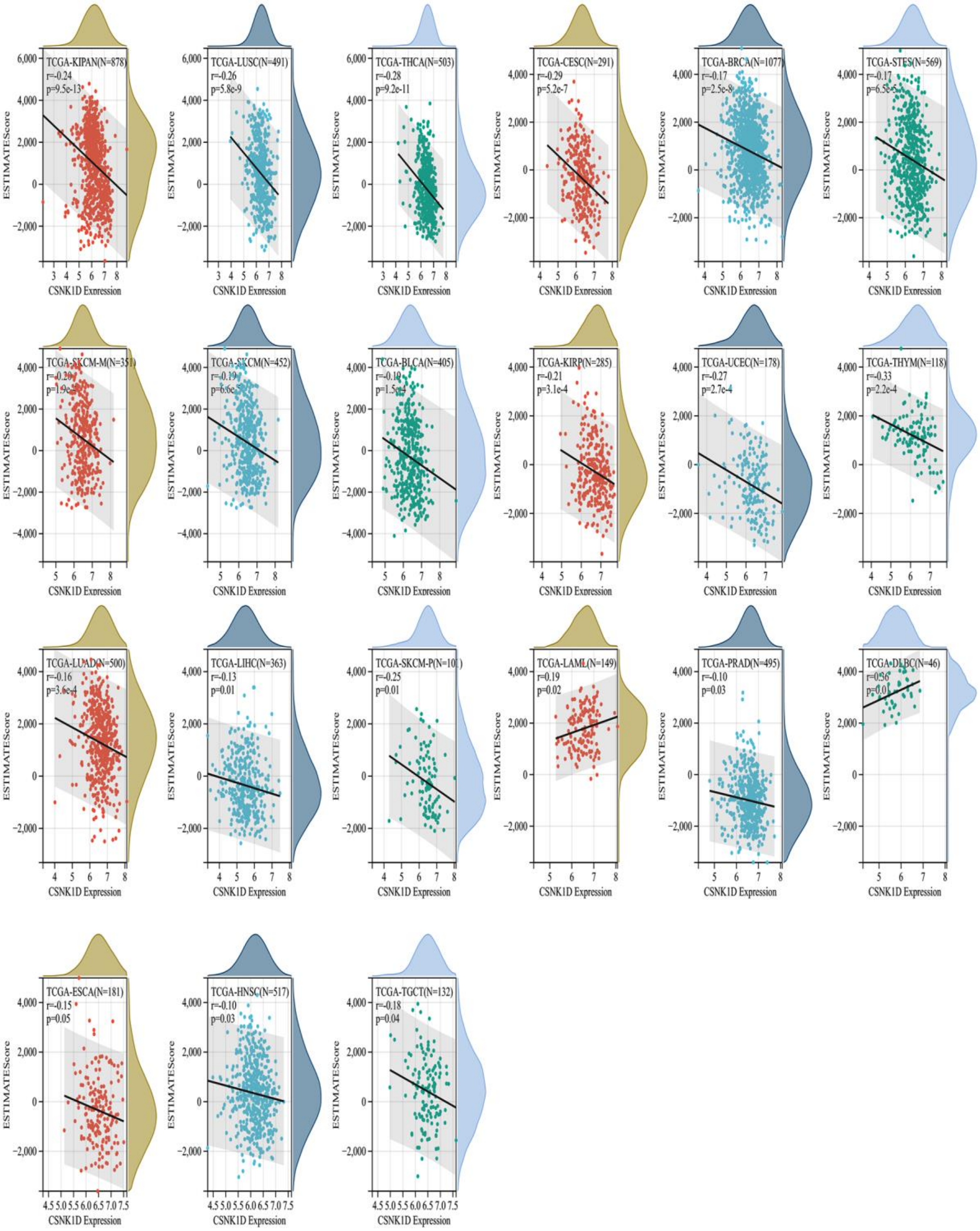

Supplementary Figure 3. Correlation of CSNK1D expression with estimate score.

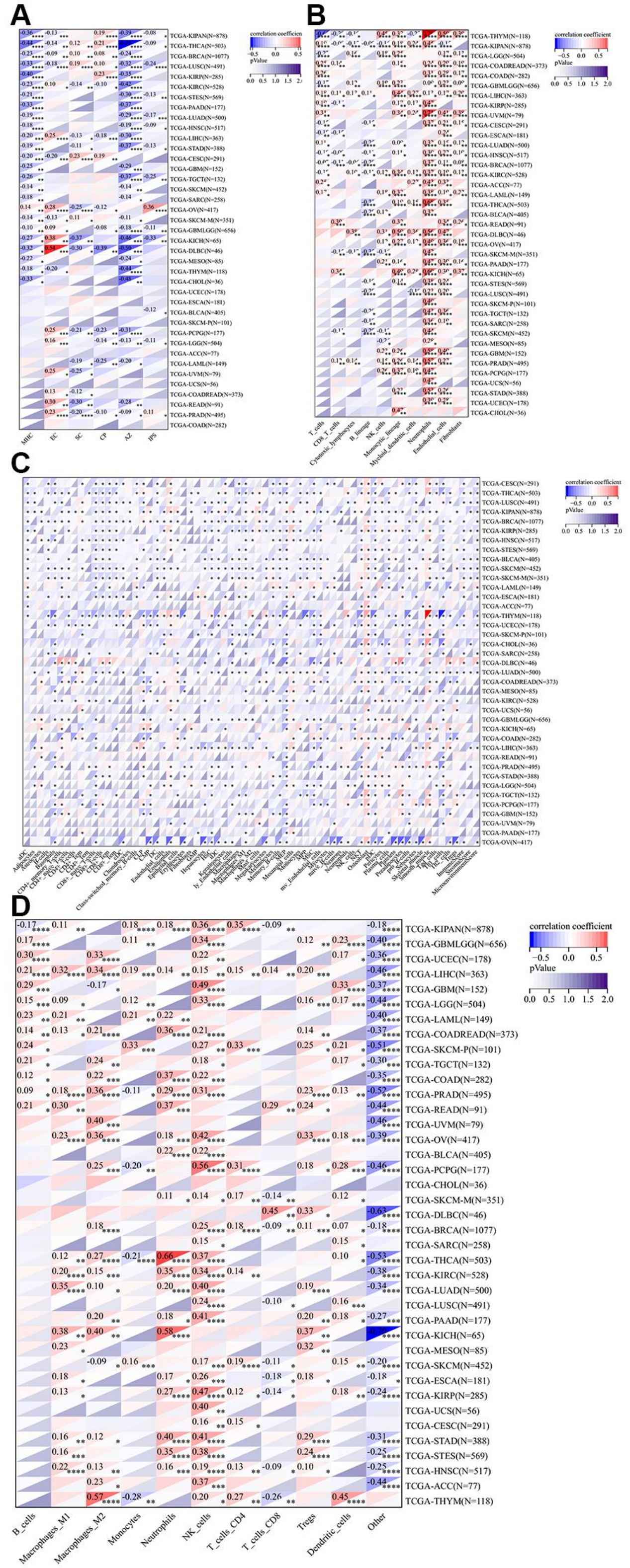

Supplementary Figure 4. Correlation analysis between the expression of CSNK1D and immune cell infiltration (**A**) The relationship between CSNK1D expression and immune cell infiltration by IPS algorithm. (**B**) The relationship between CSNK1D expression and immune cell infiltration by MCPcounter algorithm. (**C**) The relationship between CSNK1D expression and immune cell infiltration by xCell algorithm. (**D**) The relationship between CSNK1D expression and immune cell infiltration by QUANTIAEQ algorithm. $^{*}p < 0.05$; $^{**}p < 0.01$; $^{***}p < 0.001$. $^{****}p < 0.0001$.

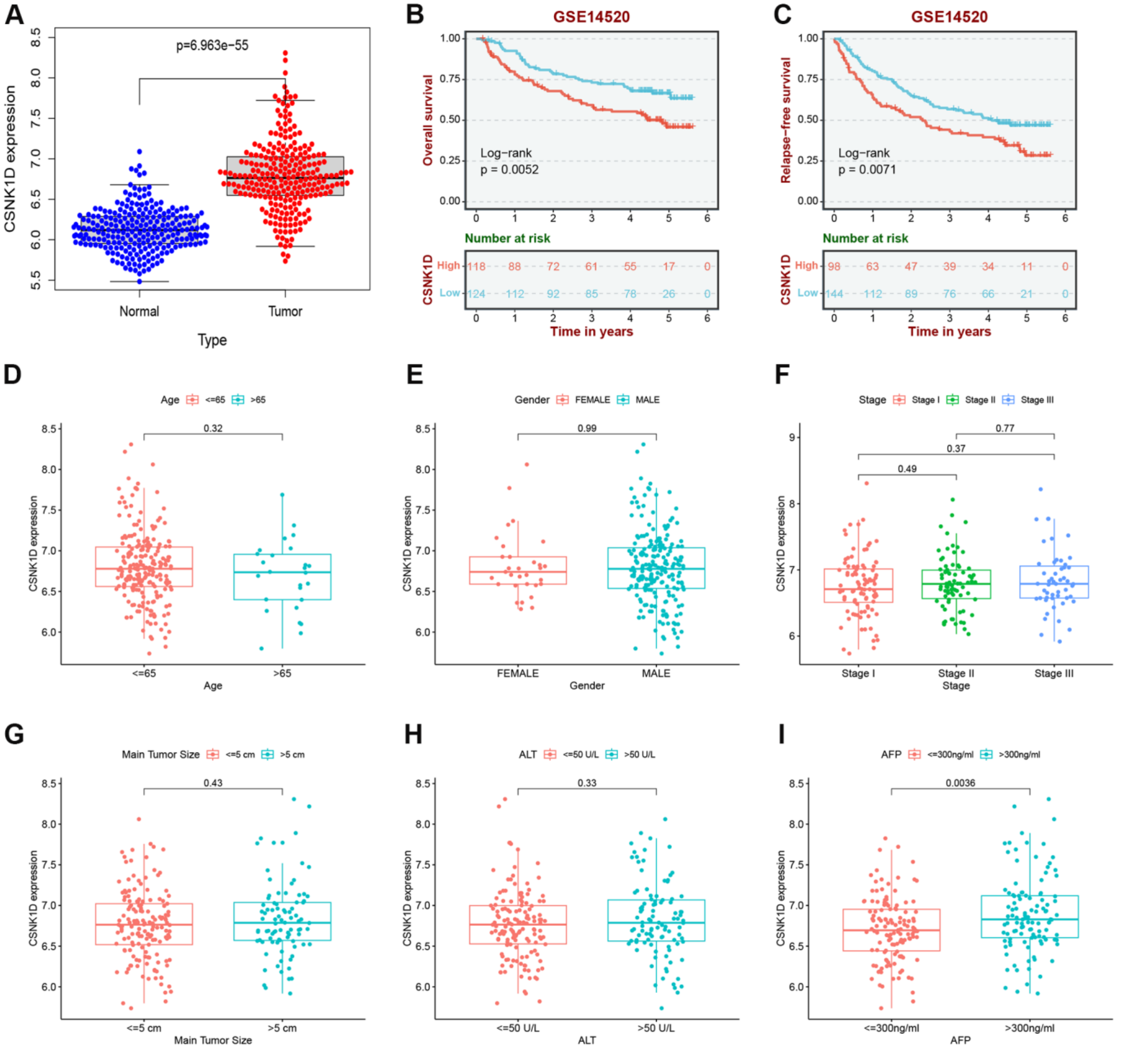

Supplementary Figure 5. Clinical correlation analysis of CSNK1D in hepatocellular carcinoma based on the GSE14520 cohort. (**A**) The mRNA expression of CSNK1D in normal and tumor. (**B**) K-M survival curves of patients with OS. (**C**) K-M survival curves of patients with RFS; Variation analysis of CSNK1D expression in different Age (**D**), Gender (**E**), stage (**F**), M stage (**E**), *N* stage (**F**), Main Tumor Size (**G**), ALT (**H**), or AFP (**I**).

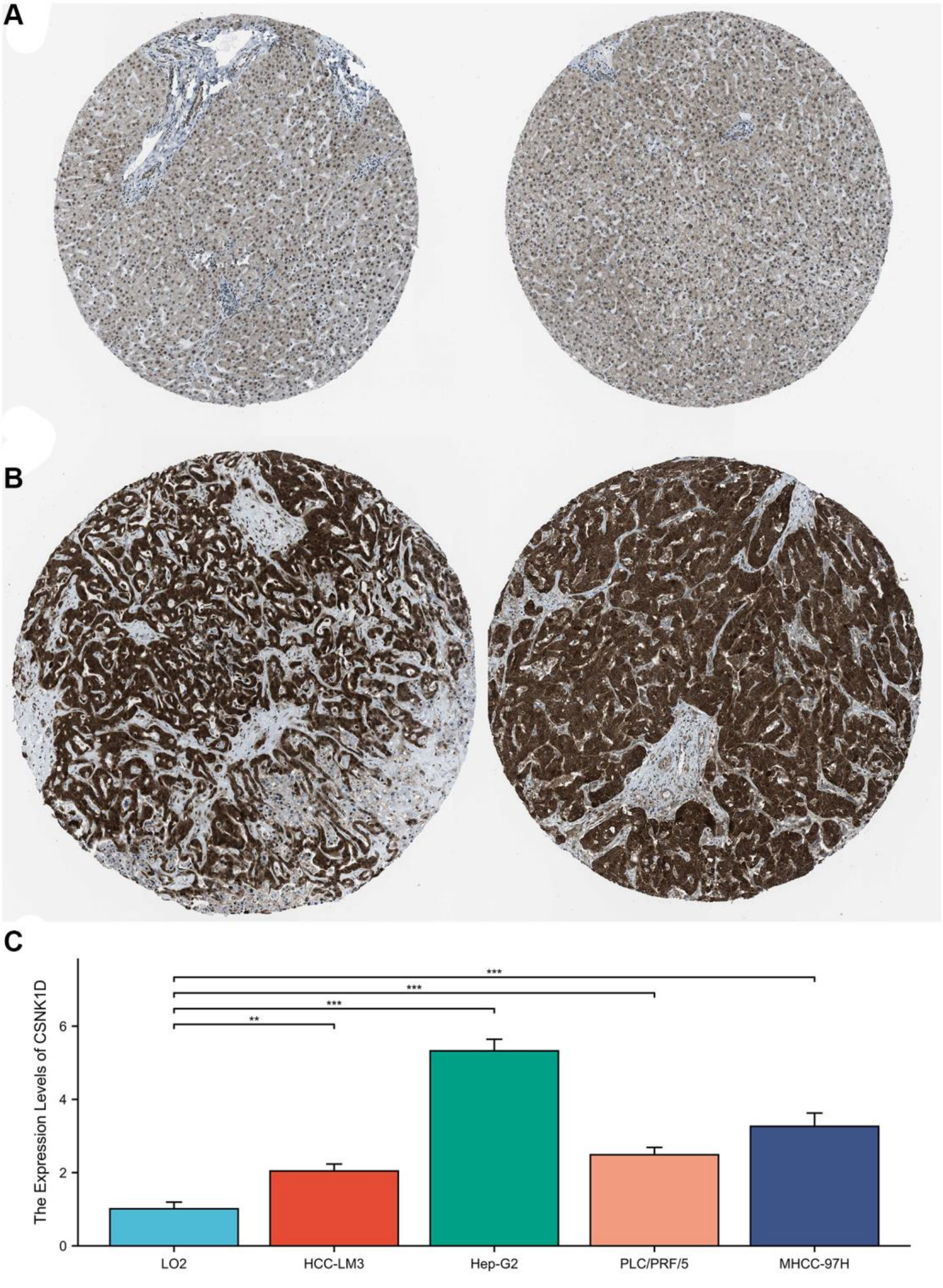

Supplementary Figure 6. Representative immunohistochemical analysis of CSNK1D in HCC by HPA database (**A**) normal tissues, (**B**) tumor tissues. (**C**) Relative mRNA expression of CSNK1D in HCC cell lines. $^{*}p < 0.05$; $^{**}p < 0.01$; $^{***}p < 0.001$.
