# Supplementary material for: Prognostic value and immunological role of CSNK1D in human cancers
Source: Aging (Albany NY). 2023 Sep 8;15(17):8948–75. doi: 10.18632/aging.205009 (PMC10522368; doi:10.18632/aging.205009)
Supplement: Supplementary Table 1 [file aging-15-205009-s002.docx]

| **Supplementary Table 1. Clinical information on the TCGA-LIHC cohort.** | | | | | | | | | |
| --- | --- | --- | --- | --- | --- | --- | --- | --- | --- |
| Id | futime | fustat | Age | Gender | Grade | Stage | T | M | N |
| TCGA-DD-A1EA | 2415 | 0 | 68 | MALE | G2 | Stage II | T2 | M0 | N0 |
| TCGA-KR-A7K0 | 65 | 1 | 65 | MALE | G1 | Stage I | T1 | M0 | N0 |
| TCGA-DD-A4NS | 2456 | 1 | 61 | FEMALE | G2 | Stage I | T1 | M0 | N0 |
| TCGA-CC-A5UC | 347 | 1 | 63 | MALE | G3 | Stage IIIA | T3 | M0 | N0 |
| TCGA-G3-AAV7 | 361 | 0 | 38 | MALE | G2 | Stage II | T2 | M0 | N0 |
| TCGA-DD-AAED | 763 | 0 | 51 | MALE | G3 | Stage I | T1 | M0 | N0 |
| TCGA-DD-AAE0 | 555 | 0 | 45 | FEMALE | G4 | Stage IIIA | T3a | M0 | N0 |
| TCGA-CC-A3M9 | 300 | 1 | 45 | MALE | G3 | Stage IIIA | T3 | M0 | N0 |
| TCGA-DD-A3A4 | 612 | 1 | 37 | MALE | G3 | Stage IIIA | T3 | M0 | N0 |
| TCGA-DD-AADB | 1242 | 0 | 51 | MALE | G4 | Stage I | T1 | M0 | N0 |
| TCGA-G3-A25V | 860 | 0 | 68 | MALE | G2 | Stage I | T1 | M0 | N0 |
| TCGA-DD-AAE7 | 644 | 0 | 72 | MALE | G2 | Stage I | T1 | M0 | N0 |
| TCGA-LG-A9QC | 425 | 0 | 48 | MALE | G2 | Stage I | T1 | M0 | NX |
| TCGA-DD-AAEH | 784 | 0 | 73 | MALE | G2 | Stage I | T1 | M0 | N0 |
| TCGA-DD-AAW1 | 1989 | 0 | 55 | MALE | G2 | Stage IIIA | T3 | M0 | N0 |
| TCGA-2Y-A9H9 | 697 | 0 | 70 | MALE | G2 | Stage I | T1 | MX | N0 |
| TCGA-5C-AAPD | 20 | 0 | 61 | MALE | G1 | Stage II | T2 | M0 | N0 |
| TCGA-EP-A2KA | 627 | 1 | 52 | FEMALE | G3 | Stage IIIA | T3a | MX | NX |
| TCGA-DD-A73B | 283 | 1 | 72 | FEMALE | G2 | Stage I | T1 | M0 | N0 |
| TCGA-UB-A7MD | 52 | 1 | 67 | MALE | G3 | Stage I | T1 | MX | N0 |
| TCGA-DD-A113 | 2425 | 0 | 55 | FEMALE | G3 | Stage II | T2 | M0 | N0 |
| TCGA-5C-A9VH | 322 | 0 | 70 | MALE | G2 | Stage I | T1 | M0 | N0 |
| TCGA-ED-A5KG | 854 | 0 | 60 | FEMALE | G2 | Stage II | T2 | M0 | N0 |
| TCGA-BC-A10U | 837 | 1 | 69 | MALE | G2 | unknow | T2 | MX | NX |
| TCGA-DD-A39V | 643 | 1 | 77 | MALE | G3 | Stage II | T2 | M0 | NX |
| TCGA-ED-A627 | 423 | 0 | 74 | MALE | G2 | Stage I | T1 | M0 | NX |
| TCGA-G3-AAV2 | 372 | 0 | 50 | MALE | G1 | Stage I | T1 | M0 | N0 |
| TCGA-ZS-A9CD | 1386 | 1 | 73 | MALE | G2 | Stage II | T2 | MX | NX |
| TCGA-GJ-A9DB | 67 | 1 | 68 | MALE | G2 | Stage I | T1 | MX | N0 |
| TCGA-G3-AAV4 | 27 | 1 | 83 | FEMALE | G1 | Stage I | T1 | M0 | N0 |
| TCGA-DD-AADP | 458 | 0 | 45 | MALE | G3 | Stage I | T1 | M0 | N0 |
| TCGA-DD-A4NL | 1711 | 0 | 46 | MALE | G1 | Stage I | T1 | M0 | N0 |
| TCGA-CC-5261 | 97 | 1 | 44 | MALE | G2 | Stage II | T2 | M0 | N0 |
| TCGA-UB-A7MC | 500 | 0 | 59 | MALE | G3 | Stage IIIA | T3a | MX | N0 |
| TCGA-CC-A7IF | 649 | 1 | 59 | MALE | G1 | Stage IIIA | T3 | M0 | N0 |
| TCGA-4R-AA8I | 262 | 1 | 66 | MALE | G2 | Stage II | T2 | MX | NX |
| TCGA-DD-A73C | 701 | 0 | 65 | FEMALE | G1 | Stage IIIA | T3a | M0 | N0 |
| TCGA-CC-5259 | 250 | 0 | 60 | FEMALE | G2 | Stage IIIC | T4 | M0 | N0 |
| TCGA-2Y-A9GW | 1271 | 1 | 64 | MALE | G2 | Stage I | T1 | MX | N0 |
| TCGA-BC-A10S | 1423 | 1 | 81 | MALE | G1 | unknow | T3 | MX | NX |
| TCGA-UB-A7ME | 486 | 0 | 51 | MALE | G2 | Stage I | T1 | MX | NX |
| TCGA-ED-A97K | 6 | 0 | 54 | MALE | G2 | Stage IIIA | T3a | M0 | N0 |
| TCGA-XR-A8TF | 693 | 1 | 74 | MALE | G1 | Stage I | T1 | MX | NX |
| TCGA-CC-A7II | 399 | 0 | 54 | MALE | G3 | Stage IIIA | T3 | M0 | N0 |
| TCGA-DD-AADQ | 436 | 0 | 59 | MALE | G3 | Stage II | T2 | M0 | N0 |
| TCGA-K7-A6G5 | 512 | 0 | 66 | MALE | G2 | Stage I | T1 | MX | N0 |
| TCGA-CC-5264 | 102 | 1 | 71 | MALE | G2 | Stage IIIA | T3 | M0 | N0 |
| TCGA-DD-A39Y | 171 | 1 | 67 | MALE | G3 | Stage I | T1 | M0 | NX |
| TCGA-ED-A8O6 | 56 | 1 | 50 | FEMALE | G3 | Stage IIIA | T3a | M0 | N0 |
| TCGA-DD-A1E9 | 2759 | 1 | 70 | MALE | G2 | Stage I | T1 | M0 | N0 |
| TCGA-DD-A1EC | 602 | 0 | 20 | FEMALE | G3 | Stage I | T1 | M0 | N0 |
| TCGA-DD-A115 | 2542 | 1 | 53 | MALE | G2 | Stage IIIA | T3 | M0 | N0 |
| TCGA-HP-A5MZ | 91 | 1 | 78 | MALE | G2 | Stage I | T1 | M0 | NX |
| TCGA-DD-A1EK | 558 | 1 | 64 | FEMALE | G2 | Stage IVB | T4 | M1 | N0 |
| TCGA-DD-AAE2 | 638 | 0 | 51 | MALE | G3 | Stage I | T1 | M0 | N0 |
| TCGA-CC-A5UE | 272 | 1 | 48 | MALE | G2 | Stage IIIB | T4 | M0 | N0 |
| TCGA-NI-A8LF | 799 | 0 | 74 | MALE | G3 | Stage I | T1 | MX | NX |
| TCGA-BW-A5NO | 20 | 0 | 50 | MALE | G2 | Stage IIIA | T3a | MX | NX |
| TCGA-CC-A5UD | 304 | 1 | 45 | MALE | G2 | Stage IIIA | T3 | M0 | N0 |
| TCGA-DD-A1EF | 394 | 1 | 57 | FEMALE | G3 | Stage I | T1 | M0 | N0 |
| TCGA-GJ-A3OU | 879 | 0 | 59 | MALE | G2 | Stage I | T1 | MX | NX |
| TCGA-DD-AAVU | 2202 | 0 | 46 | MALE | G2 | Stage II | T2 | M0 | N0 |
| TCGA-G3-A6UC | 671 | 0 | 65 | MALE | G2 | Stage IIIB | T3b | M0 | N0 |
| TCGA-CC-A9FU | 0 | 0 | 52 | FEMALE | G2 | Stage IIIA | T3a | M0 | N0 |
| TCGA-DD-AAD3 | 1295 | 0 | 43 | MALE | G2 | Stage I | T1 | M0 | N0 |
| TCGA-2Y-A9GU | 1939 | 0 | 55 | FEMALE | G2 | Stage I | T1 | MX | NX |
| TCGA-MI-A75G | 698 | 0 | 63 | MALE | G2 | Stage II | T2 | M0 | N0 |
| TCGA-FV-A4ZP | 2486 | 1 | 78 | MALE | G2 | Stage IIIA | T3 | M0 | NX |
| TCGA-PD-A5DF | 639 | 1 | 58 | FEMALE | G2 | Stage IIIB | T4 | M0 | N0 |
| TCGA-DD-A3A9 | 931 | 1 | 64 | FEMALE | G2 | Stage IVB | T4 | M1 | N0 |
| TCGA-DD-AACA | 2301 | 0 | 65 | MALE | G3 | Stage I | T1 | M0 | N0 |
| TCGA-DD-AAW2 | 1855 | 0 | 69 | MALE | G2 | Stage I | T1 | M0 | N0 |
| TCGA-DD-AADD | 1231 | 0 | 51 | MALE | G4 | Stage I | T1 | M0 | N0 |
| TCGA-XR-A8TG | 898 | 0 | 58 | MALE | G2 | Stage I | T1 | M0 | NX |
| TCGA-UB-AA0V | 314 | 0 | 69 | FEMALE | G1 | Stage I | unknow | MX | NX |
| TCGA-CC-A7IE | 217 | 1 | 57 | MALE | G2 | Stage IIIA | T3 | M0 | N0 |
| TCGA-BC-A69I | 387 | 0 | 69 | MALE | G1 | Stage I | T1 | M0 | N0 |
| TCGA-DD-AAE3 | 566 | 0 | 50 | MALE | G2 | Stage I | T1 | M0 | N0 |
| TCGA-CC-A1HT | 101 | 1 | 50 | MALE | G3 | Stage IIIA | T3 | M0 | N0 |
| TCGA-ZP-A9D1 | 21 | 0 | 56 | FEMALE | G2 | unknow | T1 | MX | NX |
| TCGA-EP-A3JL | 303 | 0 | 76 | MALE | G2 | Stage I | T1 | MX | NX |
| TCGA-CC-A7IL | 278 | 1 | 61 | MALE | G1 | Stage IIIA | T3 | M0 | N0 |
| TCGA-CC-5263 | 129 | 1 | 35 | MALE | G1 | Stage IIIA | T3 | M0 | N0 |
| TCGA-G3-A25X | 1779 | 0 | 73 | MALE | G3 | Stage II | T2 | M0 | N0 |
| TCGA-WQ-A9G7 | 30 | 0 | 71 | FEMALE | G3 | unknow | T3a | M0 | NX |
| TCGA-G3-A25Z | 655 | 0 | 58 | MALE | G2 | Stage I | T1 | M0 | N0 |
| TCGA-DD-AACX | 170 | 0 | 66 | MALE | G3 | Stage II | T2 | M0 | N0 |
| TCGA-DD-AAE6 | 141 | 0 | 59 | FEMALE | G2 | Stage I | T1 | M0 | N0 |
| TCGA-UB-AA0U | 327 | 0 | 60 | MALE | G2 | Stage II | T2 | MX | NX |
| TCGA-RG-A7D4 | 1098 | 0 | 69 | MALE | G2 | Stage II | T2 | M0 | N0 |
| TCGA-MI-A75I | 630 | 0 | 61 | MALE | G1 | unknow | T2 | MX | NX |
| TCGA-DD-AAVX | 1718 | 0 | 38 | MALE | G2 | Stage II | T2 | M0 | N0 |
| TCGA-EP-A2KC | 19 | 1 | 62 | MALE | G3 | Stage I | T1 | MX | NX |
| TCGA-DD-AADN | 898 | 0 | 59 | MALE | G4 | Stage I | T1 | MX | NX |
| TCGA-DD-A4NV | 2398 | 0 | 61 | MALE | G1 | Stage IIIA | T3 | M0 | N0 |
| TCGA-T1-A6J8 | 23 | 0 | 68 | MALE | G2 | unknow | T1 | M0 | NX |
| TCGA-DD-A4NK | 1210 | 1 | 80 | FEMALE | G2 | Stage IIIA | T3 | M0 | N0 |
| TCGA-CC-A7IJ | 382 | 0 | 56 | MALE | G3 | Stage II | T2 | M0 | N0 |
| TCGA-DD-AADR | 2028 | 0 | 58 | MALE | G3 | Stage I | T1 | M0 | N0 |
| TCGA-DD-A4NH | 917 | 0 | 65 | FEMALE | G3 | Stage IIIB | T3b | M0 | N0 |
| TCGA-BD-A2L6 | 1363 | 0 | 69 | MALE | G2 | unknow | T2 | MX | NX |
| TCGA-G3-A3CG | 673 | 0 | 80 | MALE | G2 | Stage I | T1 | M0 | N0 |
| TCGA-RC-A6M3 | 0 | 0 | 24 | MALE | G3 | Stage II | T2 | M0 | N0 |
| TCGA-DD-AACB | 2324 | 0 | 74 | FEMALE | G3 | Stage I | T1 | M0 | N0 |
| TCGA-BC-A10Y | 711 | 1 | 76 | MALE | G3 | unknow | T4 | MX | NX |
| TCGA-CC-A9FW | 248 | 0 | 68 | MALE | G2 | Stage IIIA | T3 | M0 | N0 |
| TCGA-DD-A1EL | 415 | 1 | 23 | MALE | G3 | Stage II | T2 | M0 | N0 |
| TCGA-DD-AAEE | 810 | 0 | 55 | MALE | G4 | Stage I | T1 | M0 | N0 |
| TCGA-BC-A10W | 91 | 1 | 50 | MALE | G3 | unknow | T4 | MX | NX |
| TCGA-CC-A8HU | 344 | 1 | 39 | FEMALE | G3 | Stage IIIA | T3 | M0 | N0 |
| TCGA-RC-A7SK | 472 | 0 | 59 | MALE | G3 | Stage I | T1 | M0 | N0 |
| TCGA-CC-5258 | 129 | 1 | 48 | MALE | G2 | Stage II | T2 | M0 | N0 |
| TCGA-UB-A7MF | 214 | 1 | 56 | MALE | G2 | Stage IIIA | T3a | MX | NX |
| TCGA-DD-AACL | 107 | 1 | 66 | FEMALE | G3 | Stage I | T1 | M0 | N0 |
| TCGA-BC-A10T | 837 | 1 | 76 | MALE | G1 | unknow | T4 | MX | NX |
| TCGA-DD-AACE | 2184 | 0 | 62 | MALE | G3 | Stage I | T1 | M0 | N0 |
| TCGA-BC-A110 | 2116 | 1 | 51 | FEMALE | G1 | unknow | T1 | MX | NX |
| TCGA-ED-A7PY | 390 | 0 | 20 | FEMALE | G3 | Stage II | T2 | M0 | NX |
| TCGA-DD-A4NR | 9 | 1 | 85 | FEMALE | G3 | Stage I | T1 | M0 | N0 |
| TCGA-BC-A8YO | 562 | 0 | 66 | FEMALE | G3 | Stage IIIC | T4 | M0 | N0 |
| TCGA-DD-AADU | 554 | 0 | 60 | MALE | G3 | Stage II | T2 | M0 | N0 |
| TCGA-BC-A10Z | 34 | 1 | 62 | FEMALE | G2 | Stage I | T1 | MX | N0 |
| TCGA-DD-AACK | 9 | 0 | 70 | MALE | G2 | Stage I | T1 | M0 | N0 |
| TCGA-DD-AAEK | 1067 | 0 | 51 | MALE | G3 | Stage II | T2 | M0 | N0 |
| TCGA-DD-AAW3 | 1633 | 0 | 69 | MALE | G2 | Stage I | T1 | M0 | N0 |
| TCGA-2Y-A9GZ | 848 | 1 | 82 | FEMALE | G2 | Stage II | T2 | MX | NX |
| TCGA-DD-A73F | 1085 | 0 | 77 | FEMALE | G1 | Stage I | T1 | M0 | N0 |
| TCGA-DD-AACS | 1804 | 0 | 39 | MALE | G3 | Stage I | T1 | M0 | N0 |
| TCGA-ED-A66Y | 296 | 1 | 51 | FEMALE | G3 | Stage IIIA | T3a | M0 | N0 |
| TCGA-G3-A3CJ | 594 | 0 | 52 | MALE | G2 | Stage II | T2 | M0 | N0 |
| TCGA-2Y-A9H2 | 1731 | 0 | 64 | FEMALE | G3 | Stage I | T1 | MX | N0 |
| TCGA-ED-A7PZ | 6 | 0 | 61 | MALE | G2 | Stage II | T2 | M0 | NX |
| TCGA-DD-AACI | 1618 | 0 | 69 | MALE | G3 | Stage II | T2 | M0 | N0 |
| TCGA-DD-A116 | 1622 | 1 | 68 | MALE | G3 | Stage IIIA | T3 | M0 | N0 |
| TCGA-DD-AAEG | 719 | 0 | 59 | FEMALE | G3 | Stage I | T1 | M0 | N0 |
| TCGA-DD-A114 | 1149 | 1 | 42 | MALE | G3 | Stage II | T2 | M0 | unknow |
| TCGA-ZS-A9CF | 2412 | 0 | 64 | MALE | G2 | Stage II | T2 | MX | NX |
| TCGA-RC-A6M6 | 9 | 0 | 75 | MALE | G3 | Stage II | T2 | M0 | NX |
| TCGA-5R-AA1C | 520 | 0 | 57 | MALE | G2 | Stage II | T2 | M0 | N0 |
| TCGA-CC-5262 | 103 | 1 | 67 | MALE | G1 | Stage IIIC | T4 | M0 | N0 |
| TCGA-DD-AACO | 1876 | 0 | 40 | MALE | G3 | Stage I | T1 | M0 | N0 |
| TCGA-ZP-A9CY | 782 | 0 | 66 | FEMALE | G1 | unknow | T1 | MX | NX |
| TCGA-ZP-A9D4 | 395 | 0 | 64 | FEMALE | G1 | unknow | T1 | MX | NX |
| TCGA-ZS-A9CE | 1241 | 0 | 79 | FEMALE | G1 | Stage II | T2 | MX | NX |
| TCGA-DD-AAVS | 1823 | 0 | 56 | MALE | G2 | Stage I | T1 | M0 | N0 |
| TCGA-XR-A8TC | 1339 | 0 | 43 | FEMALE | G2 | Stage I | T1 | MX | NX |
| TCGA-DD-A39W | 827 | 1 | 29 | FEMALE | G2 | Stage III | T3 | M0 | N0 |
| TCGA-BC-A69H | 444 | 0 | 64 | MALE | G3 | Stage II | T2 | M0 | NX |
| TCGA-DD-A4NA | 1008 | 0 | 67 | FEMALE | G3 | Stage IIIC | T2 | M0 | N1 |
| TCGA-MI-A75E | 507 | 0 | 61 | MALE | G2 | Stage IIIC | T4 | M0 | N0 |
| TCGA-DD-AAVZ | 1900 | 0 | 38 | MALE | G2 | Stage I | T1 | M0 | N0 |
| TCGA-G3-A5SL | 621 | 0 | 70 | MALE | G2 | Stage II | T2 | M0 | NX |
| TCGA-ED-A7XP | 400 | 0 | 53 | FEMALE | G3 | Stage II | T2 | M0 | N0 |
| TCGA-EP-A2KB | 596 | 1 | 46 | FEMALE | G2 | Stage I | T1 | MX | NX |
| TCGA-DD-A4NI | 816 | 0 | 67 | MALE | G2 | Stage II | T2 | M0 | NX |
| TCGA-CC-A9FV | 0 | 0 | 57 | MALE | G2 | Stage IIIA | T3 | M0 | N0 |
| TCGA-WQ-AB4B | 395 | 0 | 62 | MALE | G2 | Stage II | T2 | M0 | NX |
| TCGA-G3-AAV1 | 359 | 1 | 51 | MALE | G3 | Stage IIIC | T4 | M0 | N0 |
| TCGA-CC-A3MC | 363 | 0 | 54 | MALE | G2 | Stage IIIA | T3 | M0 | N0 |
| TCGA-G3-AAUZ | 480 | 0 | 48 | MALE | G2 | Stage I | T1 | M0 | N0 |
| TCGA-DD-AACM | 1769 | 0 | 48 | MALE | G3 | Stage II | T2 | M0 | N0 |
| TCGA-DD-A11D | 1560 | 1 | 57 | FEMALE | G2 | Stage I | T1 | M0 | N0 |
| TCGA-DD-AACF | 365 | 1 | 68 | MALE | G3 | Stage I | T1 | M0 | N0 |
| TCGA-BD-A3EP | 409 | 0 | 75 | FEMALE | G2 | Stage I | T1 | M0 | N0 |
| TCGA-ED-A4XI | 819 | 0 | 58 | MALE | G3 | Stage II | T2 | M0 | N0 |
| TCGA-DD-A11A | 79 | 0 | 67 | MALE | G3 | Stage I | T1 | M0 | N0 |
| TCGA-QA-A7B7 | 94 | 0 | 48 | MALE | G2 | Stage II | T2 | MX | NX |
| TCGA-DD-AADL | 636 | 0 | 58 | MALE | G4 | Stage I | T1 | M0 | N0 |
| TCGA-2Y-A9H6 | 357 | 0 | 68 | FEMALE | G2 | Stage I | T1 | MX | NX |
| TCGA-2Y-A9GY | 757 | 1 | 64 | FEMALE | G3 | Stage II | T2 | MX | NX |
| TCGA-DD-A73E | 44 | 0 | 66 | MALE | G1 | Stage I | T1 | M0 | N0 |
| TCGA-RC-A7SF | 579 | 0 | 66 | MALE | G2 | Stage I | T1 | M0 | N0 |
| TCGA-BC-A3KG | 680 | 0 | 68 | FEMALE | G3 | Stage II | T2 | M0 | N0 |
| TCGA-DD-AAVP | 2752 | 0 | 48 | MALE | G1 | Stage I | T1 | M0 | N0 |
| TCGA-ED-A459 | 910 | 0 | 47 | MALE | G2 | Stage II | T2 | M0 | N0 |
| TCGA-DD-AACW | 1424 | 0 | 43 | MALE | G3 | Stage I | T1 | M0 | N0 |
| TCGA-3K-AAZ8 | 396 | 0 | 65 | MALE | G1 | Stage IIIB | T3b | MX | NX |
| TCGA-G3-A7M5 | 447 | 0 | 76 | MALE | G2 | Stage I | T1 | MX | NX |
| TCGA-FV-A3R2 | 194 | 1 | 75 | MALE | unknow | Stage I | T1 | MX | NX |
| TCGA-BD-A3ER | 1115 | 0 | 62 | MALE | G2 | Stage II | T2 | MX | NX |
| TCGA-DD-AADY | 555 | 0 | 55 | FEMALE | G2 | Stage I | T1 | M0 | N0 |
| TCGA-DD-AACY | 1450 | 0 | 61 | MALE | G3 | Stage I | T1 | M0 | N0 |
| TCGA-DD-A1EI | 183 | 0 | 46 | MALE | G2 | Stage I | T1 | M0 | N0 |
| TCGA-2Y-A9HB | 260 | 0 | 66 | MALE | G2 | Stage I | T1 | MX | NX |
| TCGA-CC-A123 | 219 | 0 | 24 | FEMALE | G1 | Stage IIIA | T3 | M0 | N0 |
| TCGA-XR-A8TE | 925 | 0 | 16 | MALE | G1 | Stage IIIA | T3 | MX | N0 |
| TCGA-CC-A8HT | 140 | 1 | 74 | MALE | G2 | Stage IIIA | T3 | M0 | N0 |
| TCGA-G3-A5SM | 520 | 0 | 58 | MALE | G3 | Stage II | T2 | M0 | NX |
| TCGA-ES-A2HS | 688 | 1 | 80 | MALE | G2 | Stage I | T1 | MX | NX |
| TCGA-FV-A2QQ | 729 | 0 | 80 | MALE | G2 | Stage I | T1 | MX | N0 |
| TCGA-DD-AACU | 1567 | 0 | 59 | MALE | G3 | Stage I | T1 | M0 | N0 |
| TCGA-RC-A7SH | 468 | 0 | 42 | MALE | G3 | Stage II | T2 | M0 | N0 |
| TCGA-ZP-A9D2 | 765 | 1 | 51 | MALE | G2 | unknow | T2 | MX | NX |
| TCGA-DD-AACT | 1562 | 0 | 69 | FEMALE | G2 | Stage I | T1 | M0 | N0 |
| TCGA-DD-AADG | 1145 | 0 | 70 | MALE | G3 | Stage IIIA | T3a | M0 | N0 |
| TCGA-2Y-A9H0 | 3675 | 0 | 49 | MALE | G1 | Stage IIIA | T3 | M0 | N0 |
| TCGA-G3-AAV6 | 65 | 1 | 53 | FEMALE | G3 | Stage IIIA | T3a | M0 | N0 |
| TCGA-DD-A73D | 693 | 0 | 68 | FEMALE | G1 | Stage II | T2 | MX | NX |
| TCGA-FV-A2QR | 581 | 1 | 75 | MALE | G1 | Stage I | T1 | M0 | N0 |
| TCGA-DD-AADK | 1049 | 0 | 68 | FEMALE | G3 | Stage II | T2 | M0 | N0 |
| TCGA-DD-A4NE | 660 | 1 | 75 | FEMALE | G3 | Stage IIIA | T3a | M0 | N0 |
| TCGA-CC-A7IG | 299 | 1 | 47 | MALE | G2 | Stage II | T2 | M0 | N0 |
| TCGA-DD-A1EB | 2017 | 0 | 72 | FEMALE | G2 | Stage I | T1 | M0 | N0 |
| TCGA-DD-A118 | 3437 | 0 | 77 | FEMALE | G2 | Stage II | T2 | M0 | N0 |
| TCGA-5R-AAAM | 46 | 1 | 65 | FEMALE | G2 | Stage II | T2 | M0 | N0 |
| TCGA-DD-AAVQ | 2728 | 0 | 38 | MALE | G2 | Stage I | T1 | M0 | N0 |
| TCGA-G3-A25U | 1636 | 0 | 63 | FEMALE | G3 | Stage I | T1 | M0 | N0 |
| TCGA-G3-A7M8 | 430 | 0 | 31 | MALE | G1 | Stage I | T1 | MX | NX |
| TCGA-DD-A3A5 | 3125 | 1 | 66 | FEMALE | G2 | Stage III | T3 | M0 | N0 |
| TCGA-DD-AACZ | 171 | 1 | 63 | FEMALE | G4 | Stage I | T1 | M0 | N0 |
| TCGA-BC-A217 | 1397 | 1 | 75 | FEMALE | G3 | Stage II | T2 | M0 | NX |
| TCGA-ED-A7XO | 427 | 0 | 29 | MALE | G2 | Stage IIIA | T3a | M0 | N0 |
| TCGA-FV-A495 | 1 | 0 | 51 | FEMALE | G2 | Stage II | T2 | M0 | NX |
| TCGA-DD-A1EH | 1495 | 0 | 23 | MALE | G3 | Stage III | T3 | M0 | N0 |
| TCGA-FV-A23B | 1852 | 1 | 70 | FEMALE | unknow | Stage II | T2 | M0 | N0 |
| TCGA-MI-A75C | 291 | 0 | 64 | MALE | G3 | Stage I | T1 | M0 | N0 |
| TCGA-DD-A4NP | 3308 | 0 | 32 | MALE | G3 | Stage I | T1 | M0 | N0 |
| TCGA-DD-AADF | 115 | 1 | 64 | FEMALE | G4 | Stage I | T1 | M0 | N0 |
| TCGA-DD-AAEA | 575 | 0 | 65 | MALE | G3 | Stage I | T1 | M0 | N0 |
| TCGA-EP-A3RK | 363 | 0 | 73 | MALE | G2 | Stage IIIA | T3a | MX | NX |
| TCGA-BC-4072 | 1490 | 1 | 74 | FEMALE | G3 | Stage IIIA | T3 | M0 | N0 |
| TCGA-FV-A3I0 | 848 | 0 | 76 | FEMALE | G2 | Stage II | T2 | M0 | NX |
| TCGA-UB-A7MB | 601 | 0 | 24 | MALE | G3 | Stage II | T2 | MX | NX |
| TCGA-ZP-A9CZ | 706 | 0 | 72 | MALE | G1 | unknow | T1 | MX | NX |
| TCGA-MI-A75H | 747 | 0 | 77 | MALE | unknow | unknow | unknow | MX | NX |
| TCGA-G3-A5SJ | 698 | 0 | 59 | MALE | G2 | Stage I | T1 | M0 | NX |
| TCGA-DD-AAD0 | 137 | 0 | 73 | FEMALE | G2 | Stage I | T1 | M0 | N0 |
| TCGA-ED-A82E | 408 | 0 | 60 | FEMALE | G2 | Stage IIIA | T3a | M0 | N0 |
| TCGA-BC-A5W4 | 547 | 1 | 69 | MALE | G3 | Stage IIIA | T3a | M0 | NX |
| TCGA-DD-AAEI | 1531 | 0 | 72 | MALE | G2 | Stage I | T1 | M0 | N0 |
| TCGA-2Y-A9GT | 1624 | 1 | 51 | MALE | G2 | Stage I | T1 | MX | NX |
| TCGA-XR-A8TD | 1030 | 0 | 49 | FEMALE | G3 | Stage IIIB | T3 | M0 | N0 |
| TCGA-2Y-A9H4 | 1452 | 0 | 68 | MALE | G2 | Stage I | T1 | MX | N0 |
| TCGA-DD-AACD | 381 | 1 | 48 | MALE | G4 | Stage I | T1 | M0 | N0 |
| TCGA-BC-A112 | 153 | 1 | 80 | MALE | G2 | unknow | T3 | MX | NX |
| TCGA-2Y-A9GV | 2532 | 1 | 54 | FEMALE | G1 | Stage I | T1 | MX | NX |
| TCGA-DD-AADJ | 1066 | 0 | 70 | FEMALE | G3 | Stage I | T1 | M0 | N0 |
| TCGA-CC-5260 | 87 | 1 | 61 | FEMALE | G1 | Stage IIIC | T4 | M0 | N0 |
| TCGA-LG-A6GG | 387 | 0 | 79 | FEMALE | G2 | Stage II | T2 | M0 | NX |
| TCGA-G3-AAV0 | 476 | 0 | 58 | MALE | G2 | Stage I | T1 | M0 | N0 |
| TCGA-DD-AACQ | 432 | 1 | 50 | MALE | G3 | Stage II | T2 | M0 | N0 |
| TCGA-KR-A7K7 | 951 | 0 | 61 | FEMALE | G1 | Stage II | T2 | M0 | N0 |
| TCGA-DD-A4NJ | 928 | 0 | 54 | FEMALE | G2 | Stage II | T2 | M0 | N0 |
| TCGA-DD-AAVV | 2455 | 0 | 56 | MALE | G3 | Stage II | T2 | M0 | N0 |
| TCGA-DD-AAC8 | 16 | 1 | 72 | MALE | G3 | Stage I | T1 | M0 | N0 |
| TCGA-G3-A25W | 935 | 0 | 79 | FEMALE | G2 | Stage IIIB | T3b | M0 | N0 |
| TCGA-WJ-A86L | 345 | 0 | 68 | FEMALE | G2 | Stage I | T1 | MX | NX |
| TCGA-CC-A3MB | 315 | 1 | 36 | MALE | G1 | Stage IIIA | T3 | M0 | N0 |
| TCGA-G3-A7M7 | 361 | 0 | 65 | MALE | G1 | Stage I | T1 | MX | NX |
| TCGA-DD-AADC | 425 | 1 | 53 | MALE | G3 | Stage I | T1 | M0 | N0 |
| TCGA-DD-AACC | 1685 | 1 | 61 | MALE | G2 | Stage I | T1 | M0 | N0 |
| TCGA-G3-A25T | 1553 | 0 | 45 | FEMALE | G2 | Stage IIIA | T3 | M0 | N0 |
| TCGA-G3-A3CH | 780 | 0 | 53 | MALE | G2 | Stage IIIA | T3a | M0 | N0 |
| TCGA-HP-A5N0 | 752 | 1 | 90 | FEMALE | unknow | unknow | TX | M0 | NX |
| TCGA-DD-A3A7 | 419 | 1 | 67 | MALE | G3 | Stage IIIB | T3b | M0 | N0 |
| TCGA-BC-4073 | 849 | 0 | 73 | MALE | G3 | Stage IIIA | T3 | MX | N0 |
| TCGA-2Y-A9HA | 36 | 1 | 70 | MALE | G2 | Stage II | T2 | MX | NX |
| TCGA-WX-AA44 | 615 | 0 | 64 | FEMALE | G3 | Stage I | T1 | MX | NX |
| TCGA-DD-A39Z | 601 | 1 | 43 | FEMALE | G2 | Stage II | T2 | M0 | NX |
| TCGA-DD-AAD5 | 1345 | 0 | 54 | MALE | G3 | Stage I | T1 | M0 | N0 |
| TCGA-2Y-A9H7 | 1168 | 0 | 81 | FEMALE | G2 | Stage I | T1 | MX | N0 |
| TCGA-DD-A1EJ | 1005 | 1 | 71 | FEMALE | G2 | Stage IIIC | T1 | M0 | N1 |
| TCGA-DD-A4NG | 802 | 1 | 77 | MALE | G2 | Stage IIIA | T3a | M0 | NX |
| TCGA-CC-A9FS | 211 | 0 | 55 | MALE | G2 | Stage II | T2 | M0 | N0 |
| TCGA-DD-A4NO | 2245 | 0 | 65 | MALE | G1 | Stage I | T1 | M0 | N0 |
| TCGA-DD-AAVY | 1970 | 0 | 56 | MALE | G2 | Stage IIIA | T3 | M0 | N0 |
| TCGA-G3-A25Y | 452 | 1 | 52 | FEMALE | G3 | Stage I | T1 | M0 | N0 |
| TCGA-BC-A3KF | 8 | 0 | 66 | FEMALE | G2 | Stage I | T1 | M0 | NX |
| TCGA-DD-A119 | 223 | 1 | 40 | MALE | G3 | Stage IV | T3a | M1 | N0 |
| TCGA-DD-AAVR | 2513 | 0 | 44 | MALE | G2 | Stage I | T1 | M0 | N0 |
| TCGA-UB-A7MA | 848 | 0 | 62 | FEMALE | G2 | Stage II | T2b | M0 | N0 |
| TCGA-ZS-A9CG | 341 | 0 | 55 | MALE | G2 | Stage II | T2 | MX | NX |
| TCGA-ES-A2HT | 438 | 1 | 54 | MALE | G2 | Stage I | T1 | MX | NX |
| TCGA-G3-A7M9 | 56 | 1 | 70 | MALE | G2 | Stage IIIB | T3b | MX | NX |
| TCGA-DD-AADI | 1085 | 0 | 43 | FEMALE | G3 | Stage I | T1 | M0 | N0 |
| TCGA-CC-A7IK | 262 | 1 | 59 | MALE | G3 | Stage IIIA | T3 | M0 | N0 |
| TCGA-DD-AADW | 587 | 0 | 48 | MALE | G3 | Stage I | T1 | M0 | N0 |
| TCGA-FV-A4ZQ | 12 | 0 | 52 | MALE | G2 | Stage I | T1 | M0 | NX |
| TCGA-DD-AADM | 12 | 1 | 58 | MALE | G3 | Stage II | T2 | M0 | N0 |
| TCGA-DD-AAW0 | 2015 | 0 | 54 | MALE | G2 | Stage I | T1 | M0 | N0 |
| TCGA-K7-AAU7 | 359 | 0 | 61 | MALE | G2 | Stage II | T2a | MX | NX |
| TCGA-ED-A8O5 | 406 | 0 | 59 | FEMALE | G3 | Stage IIIA | T3a | M0 | N0 |
| TCGA-DD-A73G | 3478 | 0 | 73 | FEMALE | G3 | Stage I | T1 | M0 | N0 |
| TCGA-DD-A11B | 14 | 1 | 73 | MALE | G2 | Stage I | T1 | M0 | N0 |
| TCGA-WX-AA46 | 756 | 0 | 61 | MALE | G1 | Stage II | T2 | MX | NX |
| TCGA-ED-A66X | 406 | 0 | 35 | MALE | G3 | Stage IIIA | T3a | M0 | N0 |
| TCGA-BC-A10X | 770 | 1 | 52 | FEMALE | G2 | Stage IIIA | T3a | MX | N0 |
| TCGA-DD-AADS | 474 | 0 | 63 | MALE | G2 | Stage I | T1 | M0 | N0 |
| TCGA-MR-A520 | 229 | 0 | 58 | MALE | G1 | Stage I | T1 | MX | NX |
| TCGA-LG-A9QD | 366 | 0 | 68 | MALE | G2 | Stage IIIA | T3a | M0 | N0 |
| TCGA-FV-A496 | 10 | 0 | 84 | FEMALE | G2 | Stage I | T1 | M0 | NX |
| TCGA-5C-A9VG | 328 | 0 | 58 | MALE | G2 | Stage II | T2 | M0 | N0 |
| TCGA-BC-A10R | 308 | 1 | 66 | FEMALE | G2 | unknow | T3 | MX | NX |
| TCGA-DD-AA3A | 410 | 1 | 81 | FEMALE | G4 | Stage I | T1 | MX | N0 |
| TCGA-DD-A73A | 728 | 0 | 71 | MALE | G2 | Stage I | T1 | M0 | N0 |
| TCGA-BW-A5NQ | 0 | 0 | 63 | MALE | G3 | Stage I | T1 | MX | NX |
| TCGA-DD-A4NB | 989 | 0 | 25 | MALE | G2 | Stage I | T1 | M0 | N0 |
| TCGA-DD-AADO | 453 | 0 | 55 | MALE | G3 | Stage I | T1 | M0 | N0 |
| TCGA-FV-A3R3 | 366 | 1 | 38 | FEMALE | G2 | Stage I | T1 | MX | NX |
| TCGA-RC-A7SB | 588 | 0 | 53 | MALE | G2 | Stage II | T2 | M0 | N0 |
| TCGA-DD-AAD2 | 658 | 0 | 66 | MALE | G2 | Stage I | T1 | M0 | N0 |
| TCGA-DD-A1EG | 1372 | 1 | 76 | MALE | G3 | Stage I | T1 | M0 | N0 |
| TCGA-G3-A3CI | 180 | 0 | 71 | MALE | G2 | Stage I | T1 | M0 | N0 |
| TCGA-DD-AAD1 | 564 | 0 | 51 | FEMALE | G4 | Stage I | T1 | M0 | N0 |
| TCGA-DD-A11C | 662 | 0 | 69 | MALE | G3 | Stage I | T1 | M0 | N0 |
| TCGA-WX-AA47 | 556 | 1 | 33 | FEMALE | G2 | Stage IIIA | T3a | MX | NX |
| TCGA-NI-A4U2 | 1791 | 1 | 71 | MALE | G1 | Stage IIIA | T3 | MX | NX |
| TCGA-2Y-A9H3 | 1516 | 0 | 45 | MALE | G1 | Stage II | T2 | MX | NX |
| TCGA-BC-A216 | 1351 | 0 | 62 | FEMALE | G2 | Stage IIIA | T3 | M0 | NX |
| TCGA-2Y-A9H5 | 555 | 1 | 59 | FEMALE | G3 | Stage I | T1 | MX | N0 |
| TCGA-EP-A12J | 570 | 0 | 62 | MALE | G1 | Stage I | T1 | MX | NX |
| TCGA-DD-AACP | 415 | 0 | 64 | MALE | G3 | Stage I | T1 | M0 | N0 |
| TCGA-DD-A3A2 | 2131 | 1 | 76 | FEMALE | G1 | Stage I | T1 | M0 | N0 |
| TCGA-DD-A3A0 | 785 | 1 | 70 | MALE | G2 | Stage I | T1 | M0 | NX |
| TCGA-DD-A4ND | 2746 | 0 | 56 | FEMALE | G3 | Stage I | T1 | M0 | N0 |
| TCGA-G3-AAV5 | 354 | 0 | 67 | MALE | G2 | Stage II | T2 | M0 | N0 |
| TCGA-DD-AAEB | 478 | 0 | 60 | MALE | G2 | Stage I | T1 | M0 | N0 |
| TCGA-2Y-A9H8 | 633 | 1 | 85 | FEMALE | G2 | unknow | T1 | MX | NX |
| TCGA-G3-A25S | 416 | 1 | 64 | MALE | G2 | Stage I | T1 | M0 | N0 |
| TCGA-RC-A6M5 | 15 | 0 | 20 | FEMALE | G2 | Stage IVA | T1 | M0 | N1 |
| TCGA-DD-AAE4 | 608 | 0 | 49 | FEMALE | G1 | Stage I | T1 | M0 | N0 |
| TCGA-DD-AACH | 195 | 1 | 69 | MALE | G3 | Stage II | T2 | M0 | N0 |
| TCGA-DD-AAD8 | 1219 | 0 | 73 | FEMALE | G2 | Stage I | T1 | M0 | N0 |
| TCGA-CC-A7IH | 365 | 0 | 58 | MALE | G1 | Stage IIIA | T3 | M0 | N0 |
| TCGA-DD-AAE9 | 722 | 0 | 69 | MALE | G3 | Stage I | T1 | M0 | N0 |
| TCGA-DD-AADA | 1233 | 0 | 66 | FEMALE | G3 | Stage I | T1 | M0 | N0 |
| TCGA-DD-A39X | 1694 | 1 | 78 | FEMALE | G2 | Stage I | T1 | M0 | NX |
| TCGA-DD-AADE | 1202 | 0 | 50 | MALE | G4 | Stage I | T1 | M0 | N0 |
| TCGA-K7-A5RG | 519 | 0 | 66 | MALE | G1 | Stage I | T1 | MX | NX |
| TCGA-DD-AAVW | 2317 | 0 | 35 | MALE | G2 | Stage I | T1 | M0 | N0 |
| TCGA-G3-A5SI | 768 | 1 | 44 | MALE | G2 | Stage II | T2 | M0 | N0 |
| TCGA-MR-A8JO | 330 | 0 | 34 | MALE | G3 | Stage I | T1 | MX | N0 |
| TCGA-DD-AAE1 | 552 | 0 | 52 | MALE | G3 | Stage I | T1 | M0 | N0 |
| TCGA-DD-A4NN | 899 | 1 | 56 | FEMALE | G3 | Stage I | T1 | M0 | N0 |
| TCGA-BW-A5NP | 0 | 0 | 26 | FEMALE | G3 | Stage IV | T2 | M1 | N0 |
| TCGA-CC-A8HV | 279 | 1 | 51 | FEMALE | G2 | Stage II | T2 | M0 | N0 |
| TCGA-G3-A3CK | 585 | 0 | 61 | MALE | G2 | Stage I | T1 | M0 | N0 |
| TCGA-EP-A26S | 608 | 0 | 70 | MALE | G2 | Stage I | T1 | MX | N0 |
| TCGA-2Y-A9GX | 2442 | 0 | 68 | MALE | G2 | Stage I | T1 | MX | NX |
| TCGA-RC-A7S9 | 640 | 0 | 47 | FEMALE | G3 | Stage I | T1 | M0 | N0 |
| TCGA-YA-A8S7 | 412 | 1 | 68 | MALE | G3 | Stage IIIA | T3a | MX | N0 |
| TCGA-DD-A3A3 | 535 | 1 | 45 | MALE | G2 | Stage I | T1 | M0 | N0 |
| TCGA-O8-A75V | 538 | 0 | 54 | MALE | G2 | Stage I | T1 | MX | NX |
| TCGA-ZP-A9D0 | 1091 | 0 | 67 | FEMALE | G1 | unknow | T1 | MX | NX |
| TCGA-DD-A1EE | 349 | 1 | 73 | MALE | G3 | Stage IIIA | T3 | M0 | N0 |
| TCGA-DD-A3A6 | 3258 | 1 | 72 | FEMALE | G2 | Stage II | T2 | M0 | N0 |
| TCGA-RC-A6M4 | 22 | 0 | 74 | FEMALE | G2 | Stage IIIA | T3 | MX | NX |
| TCGA-DD-AAD6 | 672 | 0 | 66 | MALE | G3 | Stage IIIA | T3a | M0 | N0 |
| TCGA-K7-A5RF | 631 | 0 | 64 | MALE | G1 | Stage I | T1 | MX | NX |
| TCGA-CC-A8HS | 300 | 1 | 18 | MALE | G1 | Stage IIIC | T3 | M0 | N1 |
| TCGA-G3-A5SK | 744 | 0 | 58 | MALE | G1 | Stage I | T1 | M0 | NX |
| TCGA-DD-A4NQ | 373 | 1 | 60 | MALE | G3 | Stage II | T2 | M0 | N0 |
| TCGA-DD-A1ED | 2301 | 0 | 68 | MALE | G1 | Stage I | T1 | M0 | N0 |
| TCGA-G3-AAV3 | 412 | 0 | 58 | FEMALE | G2 | Stage II | T2 | M0 | N0 |
| TCGA-ZP-A9CV | 1088 | 1 | 59 | MALE | G1 | unknow | T1 | MX | NX |
| TCGA-DD-AACV | 1531 | 0 | 53 | MALE | G3 | Stage I | T1 | M0 | N0 |
| TCGA-DD-AACG | 469 | 1 | 52 | MALE | G4 | Stage II | T2 | M0 | N0 |
| TCGA-CC-A3MA | 303 | 1 | 61 | MALE | G2 | Stage IIIA | T3 | M0 | N0 |
| TCGA-2V-A95S | | 0 | unknow | MALE | G3 | Stage II | T2 | MX | NX |
| TCGA-2Y-A9H1 | 1229 | 1 | 58 | MALE | G2 | Stage I | T1 | MX | NX |
| TCGA-DD-A4NF | 942 | 0 | 72 | MALE | G2 | Stage I | T1 | M0 | N0 |
| TCGA-DD-A3A1 | 233 | 1 | 65 | MALE | G2 | Stage IIIA | T3b | M0 | N0 |
| TCGA-KR-A7K2 | 829 | 0 | 64 | MALE | G1 | Stage I | T1 | M0 | N0 |
| TCGA-DD-AAC9 | 347 | 0 | 51 | MALE | G2 | Stage I | T1 | M0 | N0 |
| TCGA-DD-AACJ | 2102 | 0 | 75 | MALE | G2 | Stage II | T2 | M0 | N0 |
| TCGA-DD-AACN | 1302 | 0 | 32 | MALE | G3 | Stage I | T1 | M0 | N0 |
| TCGA-GJ-A6C0 | 31 | 1 | 75 | FEMALE | G2 | Stage II | T2 | MX | NX |
| TCGA-DD-AAE8 | 664 | 0 | 45 | MALE | G3 | Stage I | T1 | M0 | N0 |
| TCGA-DD-AADV | 574 | 0 | 50 | MALE | G3 | Stage I | T1 | M0 | N0 |
| TCGA-FV-A3I1 | 247 | 1 | 81 | FEMALE | G2 | Stage II | T2 | MX | N0 |
| TCGA-BC-A10Q | 1135 | 1 | 72 | FEMALE | unknow | unknow | T2 | MX | NX |
| TCGA-2Y-A9GS | 724 | 1 | 58 | MALE | G2 | unknow | T2 | MX | NX |
| TCGA-ED-A7PX | 6 | 0 | 48 | FEMALE | G3 | Stage II | T2 | M0 | NX |
| TCGA-DD-A3A8 | 11 | 1 | 75 | MALE | G2 | Stage II | T2 | M0 | N0 |
| TCGA-G3-A7M6 | 632 | 0 | 60 | FEMALE | G3 | Stage I | T1 | MX | NX |
| TCGA-KR-A7K8 | 906 | 0 | 57 | MALE | G1 | Stage I | T1 | M0 | N0 |
| TCGA-5R-AA1D | 449 | 0 | 17 | FEMALE | G3 | Stage IIIA | T3a | M0 | N0 |
