# Supplementary material for: Prognostic value and immunological role of CSNK1D in human cancers
Source: Aging (Albany NY). 2023 Sep 8;15(17):8948–75. doi: 10.18632/aging.205009 (PMC10522368; doi:10.18632/aging.205009)
Supplement: Supplementary Table 2 [file aging-15-205009-s003.docx]

| **Supplementary Table 2. Clinical information on the ICGC-LIRI-JP cohort.** | | | |
| --- | --- | --- | --- |
| icgc_donor_id | Age | Gender | Stage |
| DO45187 | 32 | FEMALE | Stage I |
| DO50837 | 53 | FEMALE | Stage I |
| DO48721 | 58 | FEMALE | Stage I |
| DO50803 | 62 | FEMALE | Stage I |
| DO45235 | 68 | FEMALE | Stage I |
| DO45299 | 70 | FEMALE | Stage I |
| DO23532 | 71 | FEMALE | Stage I |
| DO48736 | 76 | FEMALE | Stage I |
| DO227801 | 77 | FEMALE | Stage I |
| DO23548 | 78 | FEMALE | Stage I |
| DO50791 | 79 | FEMALE | Stage I |
| DO23552 | 80 | FEMALE | Stage I |
| DO23522 | 82 | FEMALE | Stage I |
| DO45137 | 38 | MALE | Stage I |
| DO45241 | 49 | MALE | Stage I |
| DO45201 | 49 | MALE | Stage I |
| DO48737 | 52 | MALE | Stage I |
| DO45249 | 57 | MALE | Stage I |
| DO45213 | 58 | MALE | Stage I |
| DO23512 | 60 | MALE | Stage I |
| DO45105 | 61 | MALE | Stage I |
| DO50811 | 62 | MALE | Stage I |
| DO45195 | 62 | MALE | Stage I |
| DO48725 | 66 | MALE | Stage I |
| DO50807 | 67 | MALE | Stage I |
| DO23535 | 67 | MALE | Stage I |
| DO45207 | 68 | MALE | Stage I |
| DO45225 | 69 | MALE | Stage I |
| DO23550 | 69 | MALE | Stage I |
| DO50772 | 70 | MALE | Stage I |
| DO23527 | 72 | MALE | Stage I |
| DO48723 | 73 | MALE | Stage I |
| DO23540 | 73 | MALE | Stage I |
| DO45287 | 74 | MALE | Stage I |
| DO45229 | 74 | MALE | Stage I |
| DO48709 | 74 | MALE | Stage I |
| DO23547 | 74 | MALE | Stage I |
| DO50776 | 75 | MALE | Stage I |
| DO45135 | 77 | MALE | Stage I |
| DO23539 | 77 | MALE | Stage I |
| DO45247 | 31 | FEMALE | Stage II |
| DO23528 | 45 | FEMALE | Stage II |
| DO50827 | 48 | FEMALE | Stage II |
| DO45251 | 57 | FEMALE | Stage II |
| DO45227 | 57 | FEMALE | Stage II |
| DO48715 | 58 | FEMALE | Stage II |
| DO45191 | 61 | FEMALE | Stage II |
| DO48720 | 62 | FEMALE | Stage II |
| DO45107 | 63 | FEMALE | Stage II |
| DO48697 | 64 | FEMALE | Stage II |
| DO50799 | 66 | FEMALE | Stage II |
| DO45205 | 67 | FEMALE | Stage II |
| DO50774 | 68 | FEMALE | Stage II |
| DO48743 | 69 | FEMALE | Stage II |
| DO45149 | 70 | FEMALE | Stage II |
| DO48760 | 70 | FEMALE | Stage II |
| DO45203 | 71 | FEMALE | Stage II |
| DO23524 | 71 | FEMALE | Stage II |
| DO50819 | 72 | FEMALE | Stage II |
| DO45183 | 72 | FEMALE | Stage II |
| DO50789 | 73 | FEMALE | Stage II |
| DO45117 | 73 | FEMALE | Stage II |
| DO48730 | 73 | FEMALE | Stage II |
| DO48687 | 73 | FEMALE | Stage II |
| DO48728 | 74 | FEMALE | Stage II |
| DO45245 | 75 | FEMALE | Stage II |
| DO45153 | 77 | FEMALE | Stage II |
| DO45123 | 78 | FEMALE | Stage II |
| DO45179 | 78 | FEMALE | Stage II |
| DO48700 | 78 | FEMALE | Stage II |
| DO23508 | 79 | FEMALE | Stage II |
| DO23510 | 80 | FEMALE | Stage II |
| DO48727 | 81 | FEMALE | Stage II |
| DO45181 | 84 | FEMALE | Stage II |
| DO45189 | 85 | FEMALE | Stage II |
| DO45199 | 86 | FEMALE | Stage II |
| DO45239 | 42 | MALE | Stage II |
| DO50855 | 47 | MALE | Stage II |
| DO50840 | 49 | MALE | Stage II |
| DO50802 | 49 | MALE | Stage II |
| DO45253 | 50 | MALE | Stage II |
| DO48712 | 50 | MALE | Stage II |
| DO45197 | 51 | MALE | Stage II |
| DO50831 | 52 | MALE | Stage II |
| DO48761 | 55 | MALE | Stage II |
| DO50813 | 56 | MALE | Stage II |
| DO45301 | 56 | MALE | Stage II |
| DO45091 | 56 | MALE | Stage II |
| DO23536 | 56 | MALE | Stage II |
| DO45147 | 57 | MALE | Stage II |
| DO48681 | 57 | MALE | Stage II |
| DO45237 | 58 | MALE | Stage II |
| DO23534 | 60 | MALE | Stage II |
| DO23533 | 61 | MALE | Stage II |
| DO45273 | 62 | MALE | Stage II |
| DO45157 | 62 | MALE | Stage II |
| DO23517 | 62 | MALE | Stage II |
| DO50816 | 63 | MALE | Stage II |
| DO45109 | 63 | MALE | Stage II |
| DO45101 | 63 | MALE | Stage II |
| DO48689 | 63 | MALE | Stage II |
| DO23546 | 63 | MALE | Stage II |
| DO23543 | 63 | MALE | Stage II |
| DO48703 | 64 | MALE | Stage II |
| DO23514 | 64 | MALE | Stage II |
| DO50818 | 65 | MALE | Stage II |
| DO45215 | 65 | MALE | Stage II |
| DO23541 | 65 | MALE | Stage II |
| DO50822 | 66 | MALE | Stage II |
| DO45095 | 66 | MALE | Stage II |
| DO45167 | 67 | MALE | Stage II |
| DO23538 | 67 | MALE | Stage II |
| DO23545 | 67 | MALE | Stage II |
| DO23544 | 67 | MALE | Stage II |
| DO23518 | 67 | MALE | Stage II |
| DO50808 | 68 | MALE | Stage II |
| DO48751 | 68 | MALE | Stage II |
| DO50796 | 69 | MALE | Stage II |
| DO45155 | 69 | MALE | Stage II |
| DO45096 | 69 | MALE | Stage II |
| DO50825 | 70 | MALE | Stage II |
| DO48716 | 71 | MALE | Stage II |
| DO48693 | 71 | MALE | Stage II |
| DO48694 | 71 | MALE | Stage II |
| DO48691 | 71 | MALE | Stage II |
| DO45093 | 71 | MALE | Stage II |
| DO23515 | 71 | MALE | Stage II |
| DO50857 | 72 | MALE | Stage II |
| DO50834 | 72 | MALE | Stage II |
| DO50805 | 72 | MALE | Stage II |
| DO48733 | 72 | MALE | Stage II |
| DO48706 | 72 | MALE | Stage II |
| DO48679 | 72 | MALE | Stage II |
| DO45141 | 73 | MALE | Stage II |
| DO50800 | 74 | MALE | Stage II |
| DO45161 | 74 | MALE | Stage II |
| DO45111 | 74 | MALE | Stage II |
| DO45175 | 74 | MALE | Stage II |
| DO50817 | 75 | MALE | Stage II |
| DO45165 | 75 | MALE | Stage II |
| DO45295 | 76 | MALE | Stage II |
| DO45163 | 76 | MALE | Stage II |
| DO48742 | 76 | MALE | Stage II |
| DO50859 | 77 | MALE | Stage II |
| DO48695 | 77 | MALE | Stage II |
| DO45219 | 78 | MALE | Stage II |
| DO48701 | 78 | MALE | Stage II |
| DO227643 | 79 | MALE | Stage II |
| DO23537 | 79 | MALE | Stage II |
| DO50851 | 80 | MALE | Stage II |
| DO48719 | 80 | MALE | Stage II |
| DO48674 | 80 | MALE | Stage II |
| DO50804 | 81 | MALE | Stage II |
| DO45115 | 81 | MALE | Stage II |
| DO48672 | 81 | MALE | Stage II |
| DO45231 | 84 | MALE | Stage II |
| DO45185 | 89 | MALE | Stage II |
| DO50839 | 47 | FEMALE | Stage III |
| DO45303 | 62 | FEMALE | Stage III |
| DO48757 | 68 | FEMALE | Stage III |
| DO48704 | 68 | FEMALE | Stage III |
| DO45217 | 69 | FEMALE | Stage III |
| DO48684 | 71 | FEMALE | Stage III |
| DO48682 | 72 | FEMALE | Stage III |
| DO45243 | 74 | FEMALE | Stage III |
| DO50820 | 77 | FEMALE | Stage III |
| DO48692 | 77 | FEMALE | Stage III |
| DO45289 | 78 | FEMALE | Stage III |
| DO23531 | 78 | FEMALE | Stage III |
| DO45177 | 81 | FEMALE | Stage III |
| DO23511 | 83 | FEMALE | Stage III |
| DO45257 | 46 | MALE | Stage III |
| DO45099 | 46 | MALE | Stage III |
| DO45103 | 47 | MALE | Stage III |
| DO23523 | 52 | MALE | Stage III |
| DO48753 | 55 | MALE | Stage III |
| DO50809 | 56 | MALE | Stage III |
| DO45171 | 56 | MALE | Stage III |
| DO45125 | 58 | MALE | Stage III |
| DO45113 | 58 | MALE | Stage III |
| DO48677 | 58 | MALE | Stage III |
| DO45285 | 59 | MALE | Stage III |
| DO45269 | 59 | MALE | Stage III |
| DO45283 | 60 | MALE | Stage III |
| DO23509 | 60 | MALE | Stage III |
| DO45129 | 61 | MALE | Stage III |
| DO45121 | 61 | MALE | Stage III |
| DO50815 | 62 | MALE | Stage III |
| DO50783 | 62 | MALE | Stage III |
| DO48747 | 62 | MALE | Stage III |
| DO50787 | 63 | MALE | Stage III |
| DO45139 | 63 | MALE | Stage III |
| DO45173 | 63 | MALE | Stage III |
| DO23521 | 63 | MALE | Stage III |
| DO45277 | 64 | MALE | Stage III |
| DO45151 | 64 | MALE | Stage III |
| DO45133 | 64 | MALE | Stage III |
| DO48741 | 64 | MALE | Stage III |
| DO45275 | 65 | MALE | Stage III |
| DO45259 | 65 | MALE | Stage III |
| DO45159 | 65 | MALE | Stage III |
| DO23519 | 65 | MALE | Stage III |
| DO23542 | 66 | MALE | Stage III |
| DO23526 | 66 | MALE | Stage III |
| DO45279 | 67 | MALE | Stage III |
| DO45263 | 67 | MALE | Stage III |
| DO45293 | 68 | MALE | Stage III |
| DO45291 | 69 | MALE | Stage III |
| DO45143 | 69 | MALE | Stage III |
| DO45097 | 69 | MALE | Stage III |
| DO23516 | 69 | MALE | Stage III |
| DO50845 | 72 | MALE | Stage III |
| DO50832 | 73 | MALE | Stage III |
| DO45265 | 73 | MALE | Stage III |
| DO45267 | 73 | MALE | Stage III |
| DO45127 | 73 | MALE | Stage III |
| DO45309 | 73 | MALE | Stage III |
| DO48759 | 73 | MALE | Stage III |
| DO48732 | 73 | MALE | Stage III |
| DO23551 | 73 | MALE | Stage III |
| DO50850 | 74 | MALE | Stage III |
| DO50848 | 74 | MALE | Stage III |
| DO45261 | 74 | MALE | Stage III |
| DO45169 | 74 | MALE | Stage III |
| DO45119 | 74 | MALE | Stage III |
| DO23549 | 74 | MALE | Stage III |
| DO48686 | 75 | MALE | Stage III |
| DO45211 | 76 | MALE | Stage III |
| DO48746 | 76 | MALE | Stage III |
| DO45094 | 76 | MALE | Stage III |
| DO23513 | 76 | MALE | Stage III |
| DO50798 | 77 | MALE | Stage III |
| DO50829 | 78 | MALE | Stage III |
| DO45145 | 78 | MALE | Stage III |
| DO45255 | 81 | MALE | Stage III |
| DO50814 | 83 | MALE | Stage III |
| DO45233 | 83 | MALE | Stage III |
| DO45307 | 54 | FEMALE | Stage IV |
| DO45297 | 61 | FEMALE | Stage IV |
| DO45193 | 68 | FEMALE | Stage IV |
| DO50842 | 75 | FEMALE | Stage IV |
| DO45221 | 75 | FEMALE | Stage IV |
| DO50785 | 37 | MALE | Stage IV |
| DO45131 | 53 | MALE | Stage IV |
| DO23530 | 53 | MALE | Stage IV |
| DO48717 | 54 | MALE | Stage IV |
| DO23520 | 59 | MALE | Stage IV |
| DO50778 | 60 | MALE | Stage IV |
| DO45092 | 62 | MALE | Stage IV |
| DO45305 | 66 | MALE | Stage IV |
| DO45223 | 67 | MALE | Stage IV |
| DO50806 | 70 | MALE | Stage IV |
| DO50780 | 70 | MALE | Stage IV |
| DO45209 | 70 | MALE | Stage IV |
| DO23525 | 71 | MALE | Stage IV |
| DO23529 | 72 | MALE | Stage IV |
| DO50844 | 75 | MALE | Stage IV |
| DO50793 | 76 | MALE | Stage IV |
| DO45281 | 83 | MALE | Stage IV |
| DO48738 | 83 | MALE | Stage IV |
