# Supplementary material for: Prognostic value and immunological role of CSNK1D in human cancers
Source: Aging (Albany NY). 2023 Sep 8;15(17):8948–75. doi: 10.18632/aging.205009 (PMC10522368; doi:10.18632/aging.205009)
Supplement: Supplementary Table 3 [file aging-15-205009-s004.docx]

| **Supplementary Table 3. Clinical information on the GSE14520 cohort.** | | | | | | | | |
| --- | --- | --- | --- | --- | --- | --- | --- | --- |
| ID | futime | fustat | Gender | Age | ALT | Main Tumor Size | Stage | AFP |
| GSM363012 | 4.5 | 1 | MALE | 61 | <=50 U/L | >5 cm | Stage I | <=300ng/ml |
| GSM363330 | 12.6 | 1 | MALE | 50 | <=50 U/L | >5 cm | Stage I | <=300ng/ml |
| GSM363366 | 52.3 | 0 | MALE | 73 | <=50 U/L | >5 cm | Stage I | <=300ng/ml |
| GSM363039 | 52.8 | 0 | MALE | 54 | <=50 U/L | >5 cm | Stage I | <=300ng/ml |
| GSM363149 | 14.4 | 0 | MALE | 53 | <=50 U/L | >5 cm | Stage I | <=300ng/ml |
| GSM362977 | 66.3 | 0 | MALE | 41 | <=50 U/L | >5 cm | Stage I | <=300ng/ml |
| GSM363357 | 8.8 | 1 | FEMALE | 47 | <=50 U/L | >5 cm | Stage II | <=300ng/ml |
| GSM362958 | 28.2 | 1 | MALE | 56 | <=50 U/L | >5 cm | Stage II | <=300ng/ml |
| GSM363102 | 37.9 | 1 | MALE | 49 | <=50 U/L | >5 cm | Stage II | <=300ng/ml |
| GSM363311 | 19 | 0 | MALE | 77 | <=50 U/L | >5 cm | Stage II | <=300ng/ml |
| GSM363130 | 61.8 | 0 | MALE | 72 | <=50 U/L | >5 cm | Stage II | <=300ng/ml |
| GSM363313 | 36.6 | 0 | MALE | 71 | <=50 U/L | >5 cm | Stage II | <=300ng/ml |
| GSM362965 | 66.6 | 0 | MALE | 57 | <=50 U/L | >5 cm | Stage II | <=300ng/ml |
| GSM363054 | 53.8 | 0 | MALE | 51 | <=50 U/L | >5 cm | Stage II | <=300ng/ml |
| GSM363075 | 18 | 1 | FEMALE | 54 | <=50 U/L | >5 cm | Stage III | <=300ng/ml |
| GSM363017 | 12.7 | 1 | MALE | 50 | <=50 U/L | >5 cm | Stage III | <=300ng/ml |
| GSM363271 | 28.7 | 0 | MALE | 52 | <=50 U/L | >5 cm | Stage III | <=300ng/ml |
| GSM363168 | 53.8 | 0 | MALE | 51 | <=50 U/L | >5 cm | Stage III | <=300ng/ml |
| GSM363016 | 2 | 0 | MALE | 44 | <=50 U/L | >5 cm | Stage III | <=300ng/ml |
| GSM363432 | 34.4 | 1 | MALE | 41 | <=50 U/L | >5 cm | unknow | <=300ng/ml |
| GSM363034 | 45.9 | 1 | FEMALE | 30 | <=50 U/L | <=5 cm | Stage I | <=300ng/ml |
| GSM363336 | 8 | 1 | MALE | 63 | <=50 U/L | <=5 cm | Stage I | <=300ng/ml |
| GSM363104 | 35.9 | 1 | MALE | 59 | <=50 U/L | <=5 cm | Stage I | <=300ng/ml |
| GSM363269 | 52.7 | 1 | MALE | 55 | <=50 U/L | <=5 cm | Stage I | <=300ng/ml |
| GSM363364 | 27.1 | 1 | MALE | 49 | <=50 U/L | <=5 cm | Stage I | <=300ng/ml |
| GSM363378 | 31.9 | 1 | MALE | 47 | <=50 U/L | <=5 cm | Stage I | <=300ng/ml |
| GSM363176 | 50.7 | 0 | FEMALE | 65 | <=50 U/L | <=5 cm | Stage I | <=300ng/ml |
| GSM363053 | 57 | 0 | FEMALE | 57 | <=50 U/L | <=5 cm | Stage I | <=300ng/ml |
| GSM363115 | 66.6 | 0 | MALE | 74 | <=50 U/L | <=5 cm | Stage I | <=300ng/ml |
| GSM363144 | 51.1 | 0 | MALE | 72 | <=50 U/L | <=5 cm | Stage I | <=300ng/ml |
| GSM363243 | 55.8 | 0 | MALE | 69 | <=50 U/L | <=5 cm | Stage I | <=300ng/ml |
| GSM363014 | 60.7 | 0 | MALE | 61 | <=50 U/L | <=5 cm | Stage I | <=300ng/ml |
| GSM363170 | 53.3 | 0 | MALE | 59 | <=50 U/L | <=5 cm | Stage I | <=300ng/ml |
| GSM363329 | 53.2 | 0 | MALE | 56 | <=50 U/L | <=5 cm | Stage I | <=300ng/ml |
| GSM363037 | 61.2 | 0 | MALE | 54 | <=50 U/L | <=5 cm | Stage I | <=300ng/ml |
| GSM363182 | 52.7 | 0 | MALE | 50 | <=50 U/L | <=5 cm | Stage I | <=300ng/ml |
| GSM363072 | 10.4 | 0 | MALE | 49 | <=50 U/L | <=5 cm | Stage I | <=300ng/ml |
| GSM363029 | 51.3 | 0 | MALE | 48 | <=50 U/L | <=5 cm | Stage I | <=300ng/ml |
| GSM363335 | 59.4 | 0 | MALE | 45 | <=50 U/L | <=5 cm | Stage I | <=300ng/ml |
| GSM362972 | 66 | 0 | MALE | 45 | <=50 U/L | <=5 cm | Stage I | <=300ng/ml |
| GSM363083 | 53.3 | 0 | MALE | 45 | <=50 U/L | <=5 cm | Stage I | <=300ng/ml |
| GSM362970 | 67.3 | 0 | MALE | 39 | <=50 U/L | <=5 cm | Stage I | <=300ng/ml |
| GSM363202 | 60 | 0 | MALE | 33 | <=50 U/L | <=5 cm | Stage I | <=300ng/ml |
| GSM363143 | 32.6 | 1 | FEMALE | 48 | <=50 U/L | <=5 cm | Stage II | <=300ng/ml |
| GSM363267 | 47.9 | 1 | MALE | 54 | <=50 U/L | <=5 cm | Stage II | <=300ng/ml |
| GSM363362 | 5.8 | 1 | MALE | 53 | <=50 U/L | <=5 cm | Stage II | <=300ng/ml |
| GSM363355 | 9.1 | 1 | MALE | 53 | <=50 U/L | <=5 cm | Stage II | <=300ng/ml |
| GSM363186 | 7.6 | 1 | MALE | 45 | <=50 U/L | <=5 cm | Stage II | <=300ng/ml |
| GSM363038 | 15.1 | 1 | MALE | 41 | <=50 U/L | <=5 cm | Stage II | <=300ng/ml |
| GSM363309 | 53 | 1 | MALE | 34 | <=50 U/L | <=5 cm | Stage II | <=300ng/ml |
| GSM363151 | 67.1 | 0 | FEMALE | 70 | <=50 U/L | <=5 cm | Stage II | <=300ng/ml |
| GSM363344 | 38.7 | 0 | FEMALE | 52 | <=50 U/L | <=5 cm | Stage II | <=300ng/ml |
| GSM363008 | 58.4 | 0 | FEMALE | 50 | <=50 U/L | <=5 cm | Stage II | <=300ng/ml |
| GSM363082 | 57 | 0 | MALE | 64 | <=50 U/L | <=5 cm | Stage II | <=300ng/ml |
| GSM363031 | 52.8 | 0 | MALE | 58 | <=50 U/L | <=5 cm | Stage II | <=300ng/ml |
| GSM363400 | 55.6 | 0 | MALE | 58 | <=50 U/L | <=5 cm | Stage II | <=300ng/ml |
| GSM363358 | 53.9 | 0 | MALE | 56 | <=50 U/L | <=5 cm | Stage II | <=300ng/ml |
| GSM362986 | 64.5 | 0 | MALE | 55 | <=50 U/L | <=5 cm | Stage II | <=300ng/ml |
| GSM363391 | 57.3 | 0 | MALE | 55 | <=50 U/L | <=5 cm | Stage II | <=300ng/ml |
| GSM363109 | 55.2 | 0 | MALE | 50 | <=50 U/L | <=5 cm | Stage II | <=300ng/ml |
| GSM362984 | 51.4 | 0 | MALE | 49 | <=50 U/L | <=5 cm | Stage II | <=300ng/ml |
| GSM363232 | 57.9 | 0 | MALE | 49 | <=50 U/L | <=5 cm | Stage II | <=300ng/ml |
| GSM363178 | 57.3 | 0 | MALE | 42 | <=50 U/L | <=5 cm | Stage II | <=300ng/ml |
| GSM363265 | 65.1 | 0 | MALE | 41 | <=50 U/L | <=5 cm | Stage II | <=300ng/ml |
| GSM363078 | 57.9 | 1 | MALE | 47 | <=50 U/L | <=5 cm | Stage III | <=300ng/ml |
| GSM363217 | 54.5 | 0 | MALE | 58 | <=50 U/L | <=5 cm | Stage III | <=300ng/ml |
| GSM363444 | 7.3 | 1 | MALE | 46 | <=50 U/L | <=5 cm | unknow | <=300ng/ml |
| GSM363448 | 51.7 | 0 | FEMALE | 60 | <=50 U/L | <=5 cm | unknow | <=300ng/ml |
| GSM363422 | 51.6 | 0 | MALE | 67 | <=50 U/L | <=5 cm | unknow | <=300ng/ml |
| GSM363438 | 56.6 | 0 | MALE | 55 | <=50 U/L | <=5 cm | unknow | <=300ng/ml |
| GSM363424 | 63.8 | 0 | MALE | 43 | <=50 U/L | <=5 cm | unknow | <=300ng/ml |
| GSM363084 | 62.6 | 0 | MALE | 67 | >50 U/L | >5 cm | Stage I | <=300ng/ml |
| GSM363035 | 56.1 | 0 | MALE | 40 | >50 U/L | >5 cm | Stage I | <=300ng/ml |
| GSM363213 | 57.6 | 0 | MALE | 50 | >50 U/L | >5 cm | Stage II | <=300ng/ml |
| GSM363108 | 48.8 | 0 | MALE | 49 | >50 U/L | >5 cm | Stage II | <=300ng/ml |
| GSM363123 | 42.7 | 0 | MALE | 48 | >50 U/L | >5 cm | Stage II | <=300ng/ml |
| GSM363230 | 4.8 | 0 | MALE | 40 | >50 U/L | >5 cm | Stage II | <=300ng/ml |
| GSM362947 | 59.2 | 1 | MALE | 67 | >50 U/L | >5 cm | Stage III | <=300ng/ml |
| GSM363346 | 14.3 | 1 | MALE | 51 | >50 U/L | >5 cm | Stage III | <=300ng/ml |
| GSM363052 | 14.3 | 1 | MALE | 46 | >50 U/L | >5 cm | Stage III | <=300ng/ml |
| GSM363264 | 17.8 | 1 | MALE | 43 | >50 U/L | >5 cm | Stage III | <=300ng/ml |
| GSM363081 | 5.2 | 1 | MALE | 34 | >50 U/L | >5 cm | Stage III | <=300ng/ml |
| GSM363226 | 60.4 | 0 | MALE | 54 | >50 U/L | >5 cm | Stage III | <=300ng/ml |
| GSM363146 | 54.9 | 0 | MALE | 50 | >50 U/L | >5 cm | Stage III | <=300ng/ml |
| GSM363184 | 16.3 | 0 | MALE | 34 | >50 U/L | >5 cm | Stage III | <=300ng/ml |
| GSM362978 | 28.8 | 1 | MALE | 61 | >50 U/L | <=5 cm | Stage I | <=300ng/ml |
| GSM363291 | 50.1 | 1 | MALE | 54 | >50 U/L | <=5 cm | Stage I | <=300ng/ml |
| GSM363145 | 33 | 1 | MALE | 43 | >50 U/L | <=5 cm | Stage I | <=300ng/ml |
| GSM363371 | 66.3 | 0 | FEMALE | 69 | >50 U/L | <=5 cm | Stage I | <=300ng/ml |
| GSM363290 | 54.7 | 0 | FEMALE | 63 | >50 U/L | <=5 cm | Stage I | <=300ng/ml |
| GSM363388 | 57.3 | 0 | FEMALE | 58 | >50 U/L | <=5 cm | Stage I | <=300ng/ml |
| GSM363376 | 61.5 | 0 | MALE | 62 | >50 U/L | <=5 cm | Stage I | <=300ng/ml |
| GSM363222 | 48.4 | 0 | MALE | 59 | >50 U/L | <=5 cm | Stage I | <=300ng/ml |
| GSM362966 | 66.1 | 0 | MALE | 58 | >50 U/L | <=5 cm | Stage I | <=300ng/ml |
| GSM363273 | 58 | 0 | MALE | 56 | >50 U/L | <=5 cm | Stage I | <=300ng/ml |
| GSM363293 | 61.5 | 0 | MALE | 54 | >50 U/L | <=5 cm | Stage I | <=300ng/ml |
| GSM363292 | 59.9 | 0 | MALE | 53 | >50 U/L | <=5 cm | Stage I | <=300ng/ml |
| GSM363327 | 60.1 | 0 | MALE | 52 | >50 U/L | <=5 cm | Stage I | <=300ng/ml |
| GSM363192 | 52.2 | 0 | MALE | 50 | >50 U/L | <=5 cm | Stage I | <=300ng/ml |
| GSM362976 | 67 | 0 | MALE | 50 | >50 U/L | <=5 cm | Stage I | <=300ng/ml |
| GSM362964 | 67.4 | 0 | MALE | 50 | >50 U/L | <=5 cm | Stage I | <=300ng/ml |
| GSM363122 | 55.4 | 0 | MALE | 49 | >50 U/L | <=5 cm | Stage I | <=300ng/ml |
| GSM363247 | 52.9 | 0 | MALE | 44 | >50 U/L | <=5 cm | Stage I | <=300ng/ml |
| GSM363196 | 57.7 | 0 | MALE | 42 | >50 U/L | <=5 cm | Stage I | <=300ng/ml |
| GSM363348 | 14.3 | 0 | MALE | 41 | >50 U/L | <=5 cm | Stage I | <=300ng/ml |
| GSM363198 | 57.9 | 0 | MALE | 41 | >50 U/L | <=5 cm | Stage I | <=300ng/ml |
| GSM362954 | 5.5 | 0 | MALE | 40 | >50 U/L | <=5 cm | Stage I | <=300ng/ml |
| GSM362982 | 64.6 | 0 | MALE | 39 | >50 U/L | <=5 cm | Stage I | <=300ng/ml |
| GSM363172 | 6.7 | 1 | MALE | 67 | >50 U/L | <=5 cm | Stage II | <=300ng/ml |
| GSM363036 | 32.7 | 1 | MALE | 65 | >50 U/L | <=5 cm | Stage II | <=300ng/ml |
| GSM363057 | 60.5 | 1 | MALE | 63 | >50 U/L | <=5 cm | Stage II | <=300ng/ml |
| GSM363270 | 21.3 | 1 | MALE | 58 | >50 U/L | <=5 cm | Stage II | <=300ng/ml |
| GSM363239 | 42.2 | 1 | MALE | 54 | >50 U/L | <=5 cm | Stage II | <=300ng/ml |
| GSM363147 | 28.7 | 1 | MALE | 44 | >50 U/L | <=5 cm | Stage II | <=300ng/ml |
| GSM363384 | 51.6 | 1 | MALE | 43 | >50 U/L | <=5 cm | Stage II | <=300ng/ml |
| GSM363310 | 23.5 | 1 | MALE | 41 | >50 U/L | <=5 cm | Stage II | <=300ng/ml |
| GSM363128 | 30.1 | 1 | MALE | 36 | >50 U/L | <=5 cm | Stage II | <=300ng/ml |
| GSM363312 | 55.3 | 0 | FEMALE | 53 | >50 U/L | <=5 cm | Stage II | <=300ng/ml |
| GSM362987 | 60.4 | 0 | MALE | 60 | >50 U/L | <=5 cm | Stage II | <=300ng/ml |
| GSM363074 | 57.7 | 0 | MALE | 41 | >50 U/L | <=5 cm | Stage II | <=300ng/ml |
| GSM363190 | 4.8 | 0 | MALE | 41 | >50 U/L | <=5 cm | Stage II | <=300ng/ml |
| GSM362948 | 61.4 | 0 | MALE | 37 | >50 U/L | <=5 cm | Stage II | <=300ng/ml |
| GSM363105 | 9.5 | 0 | MALE | 36 | >50 U/L | <=5 cm | Stage II | <=300ng/ml |
| GSM363188 | 57.3 | 0 | MALE | 35 | >50 U/L | <=5 cm | Stage II | <=300ng/ml |
| GSM363251 | 19.6 | 1 | MALE | 47 | >50 U/L | <=5 cm | Stage III | <=300ng/ml |
| GSM363266 | 2.5 | 1 | MALE | 35 | >50 U/L | <=5 cm | Stage III | <=300ng/ml |
| GSM363275 | 59.8 | 0 | FEMALE | 67 | >50 U/L | <=5 cm | Stage III | <=300ng/ml |
| GSM362949 | 18.2 | 1 | MALE | 37 | >50 U/L | <=5 cm | unknow | <=300ng/ml |
| GSM363328 | 12 | 1 | MALE | 63 | <=50 U/L | >5 cm | Stage I | >300ng/ml |
| GSM363332 | 12.6 | 1 | MALE | 54 | <=50 U/L | >5 cm | Stage I | >300ng/ml |
| GSM363207 | 53.3 | 1 | MALE | 53 | <=50 U/L | >5 cm | Stage I | >300ng/ml |
| GSM363055 | 7.3 | 0 | FEMALE | 55 | <=50 U/L | >5 cm | Stage I | >300ng/ml |
| GSM363295 | 54.9 | 0 | MALE | 63 | <=50 U/L | >5 cm | Stage I | >300ng/ml |
| GSM363350 | 62.9 | 0 | MALE | 43 | <=50 U/L | >5 cm | Stage I | >300ng/ml |
| GSM363015 | 59.7 | 0 | MALE | 39 | <=50 U/L | >5 cm | Stage I | >300ng/ml |
| GSM363150 | 3.3 | 1 | MALE | 68 | <=50 U/L | >5 cm | Stage II | >300ng/ml |
| GSM363333 | 13.5 | 1 | MALE | 48 | <=50 U/L | >5 cm | Stage II | >300ng/ml |
| GSM363125 | 16.5 | 1 | MALE | 43 | <=50 U/L | >5 cm | Stage II | >300ng/ml |
| GSM363272 | 56.3 | 0 | MALE | 65 | <=50 U/L | >5 cm | Stage II | >300ng/ml |
| GSM363341 | 65.4 | 0 | MALE | 32 | <=50 U/L | >5 cm | Stage II | >300ng/ml |
| GSM363386 | 10 | 1 | MALE | 61 | <=50 U/L | >5 cm | Stage III | >300ng/ml |
| GSM363100 | 3.4 | 1 | MALE | 56 | <=50 U/L | >5 cm | Stage III | >300ng/ml |
| GSM363106 | 14.6 | 1 | MALE | 53 | <=50 U/L | >5 cm | Stage III | >300ng/ml |
| GSM363180 | 47.1 | 1 | MALE | 52 | <=50 U/L | >5 cm | Stage III | >300ng/ml |
| GSM363099 | 30.9 | 1 | MALE | 50 | <=50 U/L | >5 cm | Stage III | >300ng/ml |
| GSM363048 | 20.3 | 1 | MALE | 50 | <=50 U/L | >5 cm | Stage III | >300ng/ml |
| GSM363080 | 2.3 | 1 | MALE | 49 | <=50 U/L | >5 cm | Stage III | >300ng/ml |
| GSM363218 | 15.2 | 1 | MALE | 48 | <=50 U/L | >5 cm | Stage III | >300ng/ml |
| GSM363404 | 12 | 1 | MALE | 46 | <=50 U/L | >5 cm | Stage III | >300ng/ml |
| GSM363071 | 13.8 | 1 | MALE | 35 | <=50 U/L | >5 cm | Stage III | >300ng/ml |
| GSM363129 | 3.5 | 1 | MALE | 34 | <=50 U/L | >5 cm | Stage III | >300ng/ml |
| GSM363297 | 3 | 0 | FEMALE | 58 | <=50 U/L | >5 cm | Stage III | >300ng/ml |
| GSM363126 | 59.5 | 0 | MALE | 65 | <=50 U/L | >5 cm | Stage III | >300ng/ml |
| GSM363166 | 53.8 | 0 | MALE | 59 | <=50 U/L | >5 cm | Stage III | >300ng/ml |
| GSM363032 | 24.9 | 0 | MALE | 52 | <=50 U/L | >5 cm | Stage III | >300ng/ml |
| GSM363051 | 20.6 | 0 | MALE | 43 | <=50 U/L | >5 cm | Stage III | >300ng/ml |
| GSM363235 | 32.8 | 0 | MALE | 41 | <=50 U/L | >5 cm | Stage III | >300ng/ml |
| GSM363428 | 3.8 | 1 | MALE | 76 | <=50 U/L | >5 cm | unknow | >300ng/ml |
| GSM363426 | 1.8 | 1 | MALE | 33 | <=50 U/L | >5 cm | unknow | >300ng/ml |
| GSM363446 | 52.1 | 0 | MALE | 50 | <=50 U/L | >5 cm | unknow | >300ng/ml |
| GSM363107 | 7 | 1 | FEMALE | 71 | <=50 U/L | <=5 cm | Stage I | >300ng/ml |
| GSM363033 | 36.4 | 1 | MALE | 63 | <=50 U/L | <=5 cm | Stage I | >300ng/ml |
| GSM363337 | 65.5 | 0 | FEMALE | 53 | <=50 U/L | <=5 cm | Stage I | >300ng/ml |
| GSM362988 | 51.1 | 0 | FEMALE | 52 | <=50 U/L | <=5 cm | Stage I | >300ng/ml |
| GSM363209 | 60.8 | 0 | FEMALE | 45 | <=50 U/L | <=5 cm | Stage I | >300ng/ml |
| GSM363316 | 54.7 | 0 | FEMALE | 35 | <=50 U/L | <=5 cm | Stage I | >300ng/ml |
| GSM363343 | 65.1 | 0 | FEMALE | 30 | <=50 U/L | <=5 cm | Stage I | >300ng/ml |
| GSM363164 | 55.2 | 0 | FEMALE | 27 | <=50 U/L | <=5 cm | Stage I | >300ng/ml |
| GSM363360 | 53.3 | 0 | MALE | 71 | <=50 U/L | <=5 cm | Stage I | >300ng/ml |
| GSM363205 | 58 | 0 | MALE | 70 | <=50 U/L | <=5 cm | Stage I | >300ng/ml |
| GSM363224 | 57.9 | 0 | MALE | 70 | <=50 U/L | <=5 cm | Stage I | >300ng/ml |
| GSM362952 | 54.3 | 0 | MALE | 61 | <=50 U/L | <=5 cm | Stage I | >300ng/ml |
| GSM363268 | 56 | 0 | MALE | 56 | <=50 U/L | <=5 cm | Stage I | >300ng/ml |
| GSM363288 | 60.3 | 0 | MALE | 56 | <=50 U/L | <=5 cm | Stage I | >300ng/ml |
| GSM363298 | 53.6 | 0 | MALE | 52 | <=50 U/L | <=5 cm | Stage I | >300ng/ml |
| GSM363101 | 56.3 | 0 | MALE | 50 | <=50 U/L | <=5 cm | Stage I | >300ng/ml |
| GSM362993 | 3.8 | 0 | MALE | 50 | <=50 U/L | <=5 cm | Stage I | >300ng/ml |
| GSM363215 | 18.6 | 0 | MALE | 48 | <=50 U/L | <=5 cm | Stage I | >300ng/ml |
| GSM363289 | 54.2 | 0 | MALE | 46 | <=50 U/L | <=5 cm | Stage I | >300ng/ml |
| GSM362950 | 57.5 | 0 | MALE | 45 | <=50 U/L | <=5 cm | Stage I | >300ng/ml |
| GSM363087 | 54 | 0 | MALE | 40 | <=50 U/L | <=5 cm | Stage I | >300ng/ml |
| GSM363077 | 52.7 | 0 | MALE | 40 | <=50 U/L | <=5 cm | Stage I | >300ng/ml |
| GSM363296 | 27.5 | 1 | FEMALE | 52 | <=50 U/L | <=5 cm | Stage II | >300ng/ml |
| GSM363011 | 17.6 | 1 | MALE | 57 | <=50 U/L | <=5 cm | Stage II | >300ng/ml |
| GSM363127 | 22.2 | 1 | MALE | 55 | <=50 U/L | <=5 cm | Stage II | >300ng/ml |
| GSM363073 | 26.5 | 1 | MALE | 41 | <=50 U/L | <=5 cm | Stage II | >300ng/ml |
| GSM363200 | 23.9 | 1 | MALE | 26 | <=50 U/L | <=5 cm | Stage II | >300ng/ml |
| GSM363249 | 59.2 | 0 | FEMALE | 60 | <=50 U/L | <=5 cm | Stage II | >300ng/ml |
| GSM363314 | 57.1 | 0 | FEMALE | 48 | <=50 U/L | <=5 cm | Stage II | >300ng/ml |
| GSM363098 | 56.9 | 0 | MALE | 60 | <=50 U/L | <=5 cm | Stage II | >300ng/ml |
| GSM362994 | 54.8 | 0 | MALE | 49 | <=50 U/L | <=5 cm | Stage II | >300ng/ml |
| GSM363086 | 60.5 | 0 | MALE | 44 | <=50 U/L | <=5 cm | Stage II | >300ng/ml |
| GSM363294 | 62.6 | 0 | MALE | 25 | <=50 U/L | <=5 cm | Stage II | >300ng/ml |
| GSM712542 | 7.5 | 1 | MALE | 61 | <=50 U/L | <=5 cm | Stage III | >300ng/ml |
| GSM363393 | 4 | 1 | MALE | 46 | <=50 U/L | <=5 cm | Stage III | >300ng/ml |
| GSM363420 | 7.3 | 1 | MALE | 72 | <=50 U/L | <=5 cm | unknow | >300ng/ml |
| GSM363049 | 62.4 | 0 | MALE | 45 | <=50 U/L | unknow | Stage I | >300ng/ml |
| GSM363354 | 10.4 | 1 | MALE | 66 | <=50 U/L | >5 cm | Stage I | >300ng/ml |
| GSM363331 | 36.5 | 1 | FEMALE | 54 | >50 U/L | >5 cm | Stage I | >300ng/ml |
| GSM363237 | 57.5 | 0 | MALE | 69 | >50 U/L | >5 cm | Stage I | >300ng/ml |
| GSM363241 | 54.2 | 0 | MALE | 55 | >50 U/L | >5 cm | Stage I | >300ng/ml |
| GSM363009 | 58.9 | 0 | MALE | 50 | >50 U/L | >5 cm | Stage II | >300ng/ml |
| GSM363211 | 8.9 | 1 | MALE | 61 | >50 U/L | >5 cm | Stage III | >300ng/ml |
| GSM363204 | 14.1 | 1 | MALE | 53 | >50 U/L | >5 cm | Stage III | >300ng/ml |
| GSM363079 | 7.8 | 1 | MALE | 53 | >50 U/L | >5 cm | Stage III | >300ng/ml |
| GSM363085 | 54.8 | 1 | MALE | 49 | >50 U/L | >5 cm | Stage III | >300ng/ml |
| GSM363263 | 3 | 1 | MALE | 39 | >50 U/L | >5 cm | Stage III | >300ng/ml |
| GSM363152 | 8.3 | 1 | MALE | 39 | >50 U/L | >5 cm | Stage III | >300ng/ml |
| GSM363076 | 23 | 1 | MALE | 35 | >50 U/L | >5 cm | Stage III | >300ng/ml |
| GSM363069 | 13.6 | 1 | MALE | 34 | >50 U/L | >5 cm | Stage III | >300ng/ml |
| GSM363220 | 54.2 | 0 | MALE | 59 | >50 U/L | >5 cm | Stage III | >300ng/ml |
| GSM363245 | 57.5 | 0 | MALE | 47 | >50 U/L | >5 cm | Stage III | >300ng/ml |
| GSM363169 | 39.4 | 0 | MALE | 45 | >50 U/L | >5 cm | Stage III | >300ng/ml |
| GSM363436 | 3.8 | 1 | MALE | 48 | >50 U/L | >5 cm | unknow | >300ng/ml |
| GSM363440 | 55.5 | 0 | MALE | 73 | >50 U/L | >5 cm | unknow | >300ng/ml |
| GSM363070 | 57 | 0 | MALE | 50 | >50 U/L | >5 cm | unknow | >300ng/ml |
| GSM362971 | 48.1 | 1 | MALE | 52 | >50 U/L | <=5 cm | Stage I | >300ng/ml |
| GSM362959 | 9.5 | 1 | MALE | 45 | >50 U/L | <=5 cm | Stage I | >300ng/ml |
| GSM363326 | 37.2 | 1 | MALE | 37 | >50 U/L | <=5 cm | Stage I | >300ng/ml |
| GSM363317 | 62.8 | 0 | MALE | 58 | >50 U/L | <=5 cm | Stage I | >300ng/ml |
| GSM362983 | 64.2 | 0 | MALE | 32 | >50 U/L | <=5 cm | Stage I | >300ng/ml |
| GSM363056 | 19.2 | 1 | FEMALE | 59 | >50 U/L | <=5 cm | Stage II | >300ng/ml |
| GSM363013 | 7.7 | 1 | MALE | 58 | >50 U/L | <=5 cm | Stage II | >300ng/ml |
| GSM363274 | 26.9 | 1 | MALE | 53 | >50 U/L | <=5 cm | Stage II | >300ng/ml |
| GSM363352 | 16.2 | 1 | MALE | 51 | >50 U/L | <=5 cm | Stage II | >300ng/ml |
| GSM363142 | 46.1 | 1 | MALE | 28 | >50 U/L | <=5 cm | Stage II | >300ng/ml |
| GSM363339 | 65.4 | 0 | FEMALE | 49 | >50 U/L | <=5 cm | Stage II | >300ng/ml |
| GSM363148 | 52.4 | 0 | MALE | 64 | >50 U/L | <=5 cm | Stage II | >300ng/ml |
| GSM363174 | 67.1 | 0 | MALE | 48 | >50 U/L | <=5 cm | Stage II | >300ng/ml |
| GSM363194 | 4.5 | 0 | MALE | 44 | >50 U/L | <=5 cm | Stage II | >300ng/ml |
| GSM363315 | 65.4 | 0 | MALE | 43 | >50 U/L | <=5 cm | Stage II | >300ng/ml |
| GSM363050 | 54.2 | 0 | MALE | 32 | >50 U/L | <=5 cm | Stage II | >300ng/ml |
| GSM362960 | 66.1 | 0 | MALE | 21 | >50 U/L | <=5 cm | Stage II | >300ng/ml |
| GSM363030 | 4.5 | 1 | MALE | 58 | >50 U/L | <=5 cm | Stage III | >300ng/ml |
| GSM363121 | 51.2 | 0 | MALE | 39 | >50 U/L | <=5 cm | Stage III | >300ng/ml |
| GSM363368 | 15.4 | 1 | MALE | 66 | >50 U/L | <=5 cm | unknow | >300ng/ml |
| GSM363430 | 3.7 | 1 | MALE | 47 | >50 U/L | <=5 cm | unknow | >300ng/ml |
| GSM363442 | 4.6 | 1 | MALE | 42 | >50 U/L | <=5 cm | unknow | >300ng/ml |
| GSM363124 | 53.5 | 0 | MALE | 67 | <=50 U/L | <=5 cm | Stage I | unknow |
| GSM362992 | 64.3 | 0 | FEMALE | 41 | >50 U/L | >5 cm | Stage I | unknow |
| GSM363010 | 62 | 0 | MALE | 47 | >50 U/L | >5 cm | Stage I | unknow |
| GSM362956 | 13.3 | 1 | MALE | 33 | >50 U/L | >5 cm | Stage III | unknow |
